# Correction: Functional redundancy and formin-isoform independent localization of tropomyosin paralogs in Saccharomyces cerevisiae

**DOI:** 10.1371/journal.pgen.1012065

**Published:** 2026-04-28

**Authors:** The PLOS Genetics Staff

## Notice of Republication

This article was republished on January 21, 2026, to correct an error in the XML. The author V.T. Bagyashree was listed with incorrect author contributions in the XML. The publisher apologises for this error.
