# Supplementary figures and images for: Functional redundancy and formin-isoform independent localization of tropomyosin paralogs in Saccharomyces cerevisiae
Source: PLoS Genet. 2025 Sep 9;21(9):e1011859. doi: 10.1371/journal.pgen.1011859 (PMC12440208; doi:10.1371/journal.pgen.1011859)

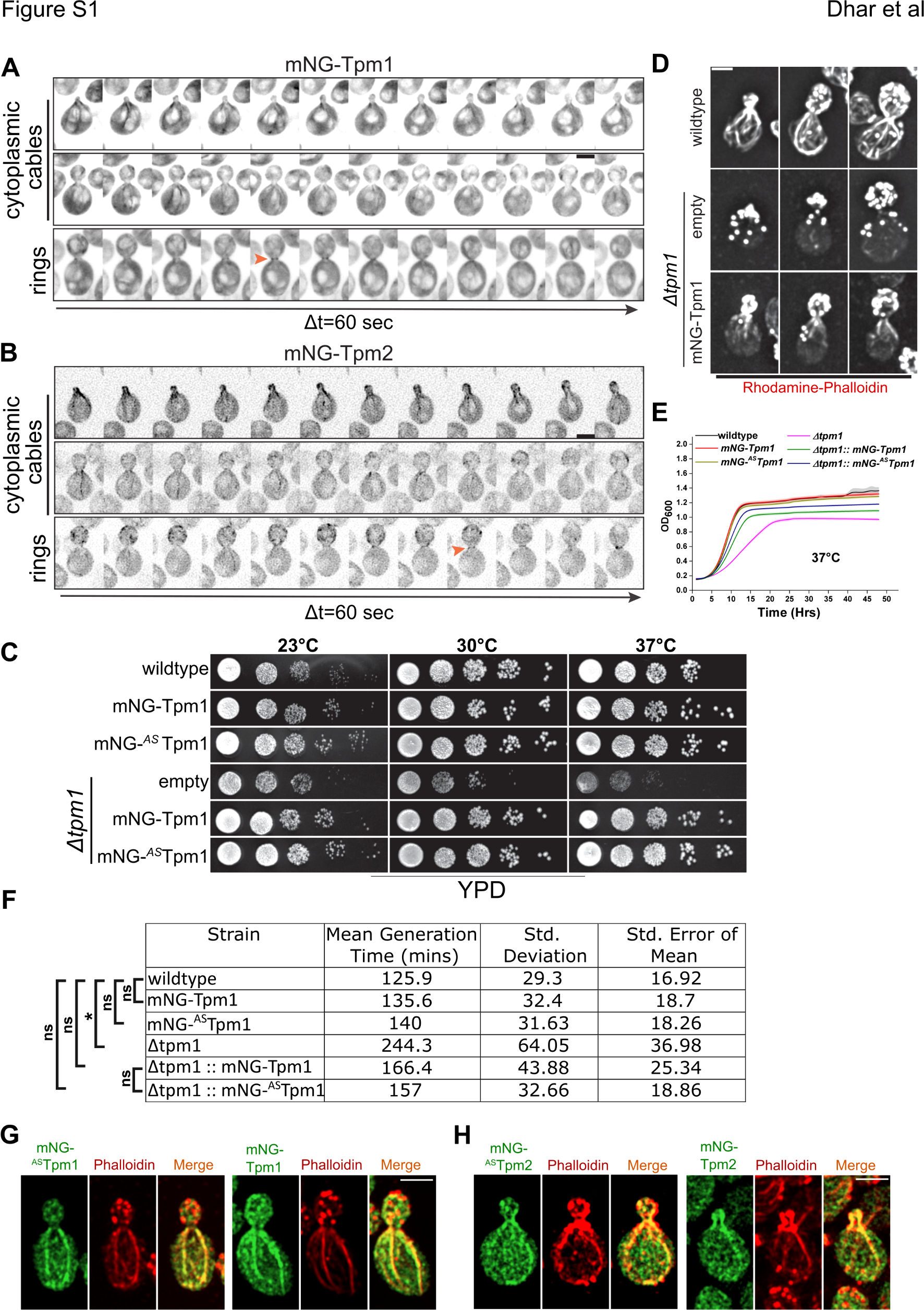

Supplement: S1 Fig — (A) Representative time-lapse montages of wildtype yeast cells expressing mNG-Tpm1; scale bar - 3μm. (B) Representative time-lapse montages of wildtype yeast cells expressing mNG-Tpm2; scale bar - 3μm. (C) Spot assay images for indicated yeast strains performed at 23°C, 30°C, and 37°C. (D) Representative images of cells of indicated yeast strains stained with Rhodamine-phalloidin; scale bar – 2μm. (E) Plot representing growth curves of indicated yeast strains performed at 37°C. y-axis represents mean absorbance at 600nm. (F) Table showing descriptive statistics of Generation Time derived from analysis of growth curve experiment represented in (E). (G-H) Representative images of cells expressing indicated mNeonGreen-Tpm fusion proteins stained with Rhodamine-phalloidin; scale bar - 2μm. (TIF) [file pgen.1011859.s001.tif]

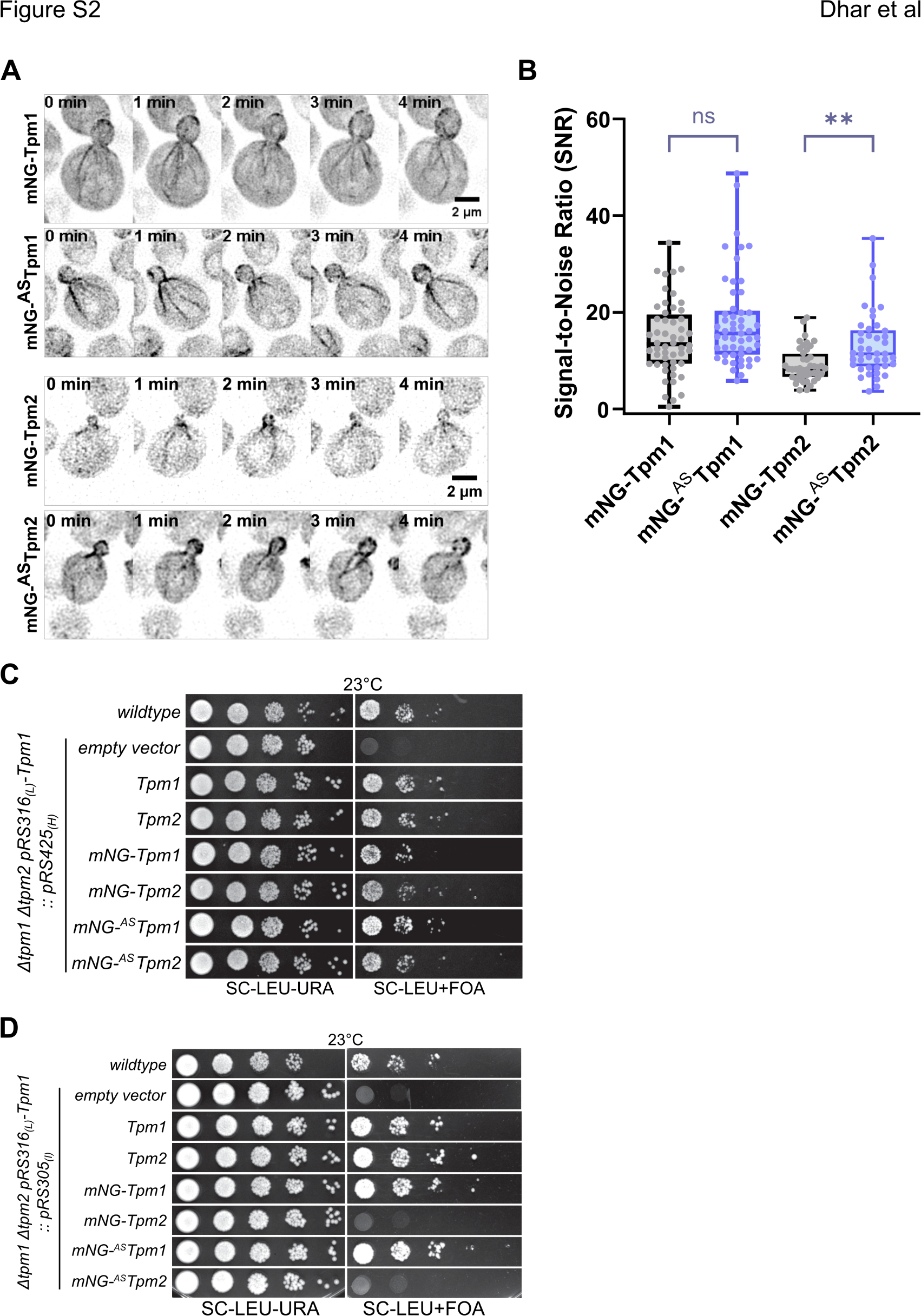

Supplement: S2 Fig — (A) Representative time-lapse images acquired at identical settings showing localization of indicated mNG-Tpm variants in wildtype cells. (B) Plot depicting Signal-to-Noise Ratio of mNG-Tpm fluorescence on cables from images shown in (A); n ≥ 42 cables per strain. (C-D) Spot assay images for indicated yeast strains performed at 23°C, 30°C, and 37°C to test for rescue of synthetic lethality of Tpm1 and Tpm2. (TIF) [file pgen.1011859.s002.tif]

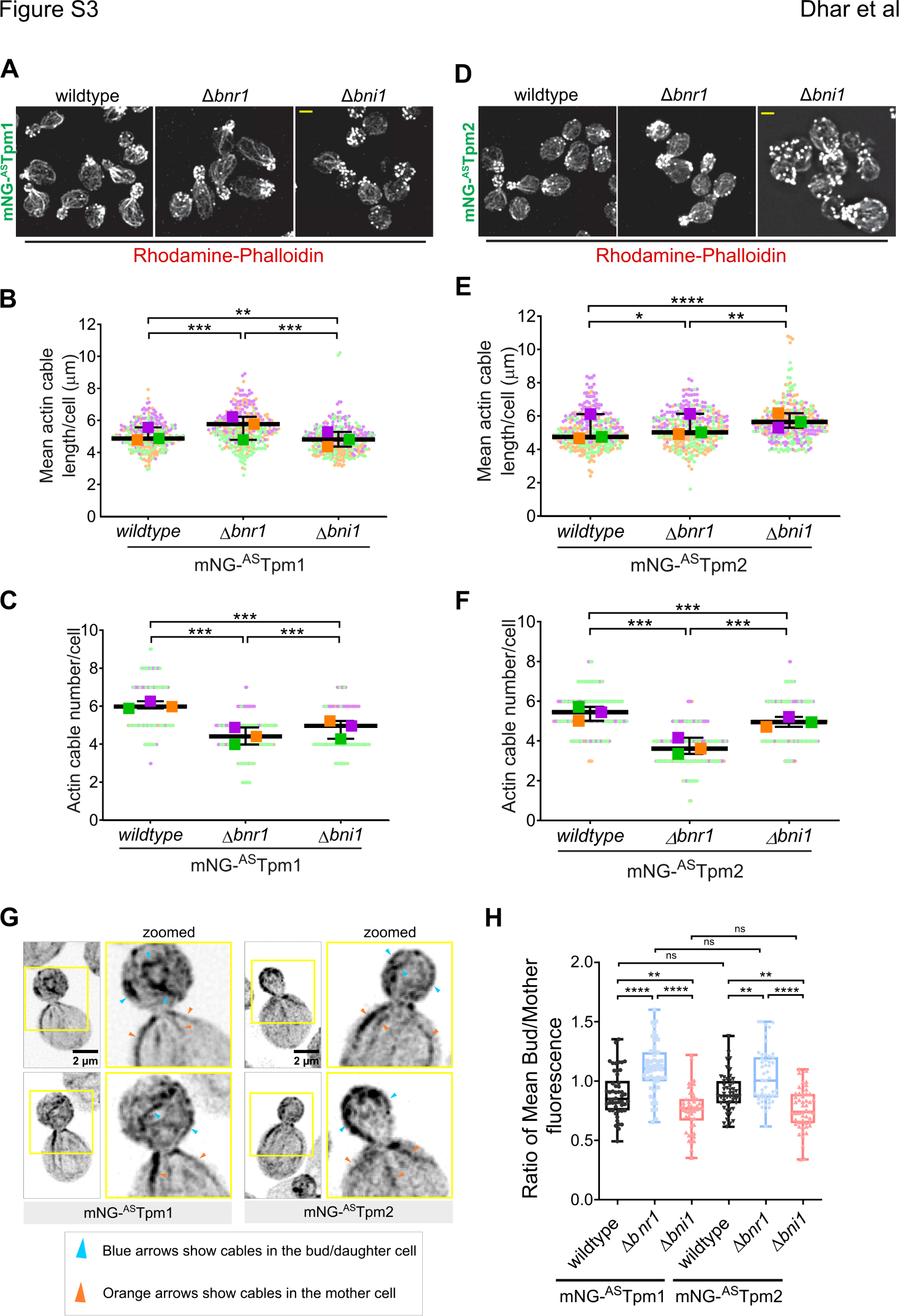

Supplement: S3 Fig — (A) Representative images of cells of wildtype, Δbnr1, and Δbni1 cells expressing mNG-ASTpm1 stained with Rhodamine-phalloidin; scale bar - 2μm. (B) Superplot representing mean actin cable length per cell in wildtype, Δbnr1, and Δbni1 cells expressing mNG-ASTpm1; n = 100 cells per strain per replicate, N = 3. (C) Superplot representing mean actin cable number per cell in wildtype, Δbnr1, and Δbni1 cells expressing mNG-ASTpm1; n = 100 per strain per replicate, N = 3. (D) Representative images of cells of wildtype, Δbnr1, and Δbni1 cells expressing mNG-ASTpm2 stained with Rhodamine-phalloidin; scale bar – 2μm. (E) Superplot representing mean actin cable length per cell in wildtype, Δbnr1, and Δbni1 cells expressing mNG-ASTpm2; n = 100 cells per strain per replicate, N = 3. (F) Superplot representing mean actin cable number per cell in wildtype, Δbnr1, and Δbni1 cells expressing mNG-ASTpm2; n = 100 cells per strain per replicate, N = 3. (G) Representative images depicting localization of mNG-ASTpm fusion proteins to actin cables in both and bud and mother cell compartment in wildtype cells. (H) Box and whiskers plot showing ratio of mean mNG fluorescence in the bud to the mother compartment in the indicated strains; n = 50 cells per strain. Box represents 25th and 75th percentile, line represents median, whiskers represent minimum and maximum value. (Superplots represent datapoints and means from three independent biological replicates marked in different colours; Kruskal-Wallis test with Dunn’s Multiple Comparisons test was used in (B), (C), (E), (F); * p < 0.05, ** p < 0.01, *** p < 0.001, **** p < 0.0001) (TIF) [file pgen.1011859.s003.tif]

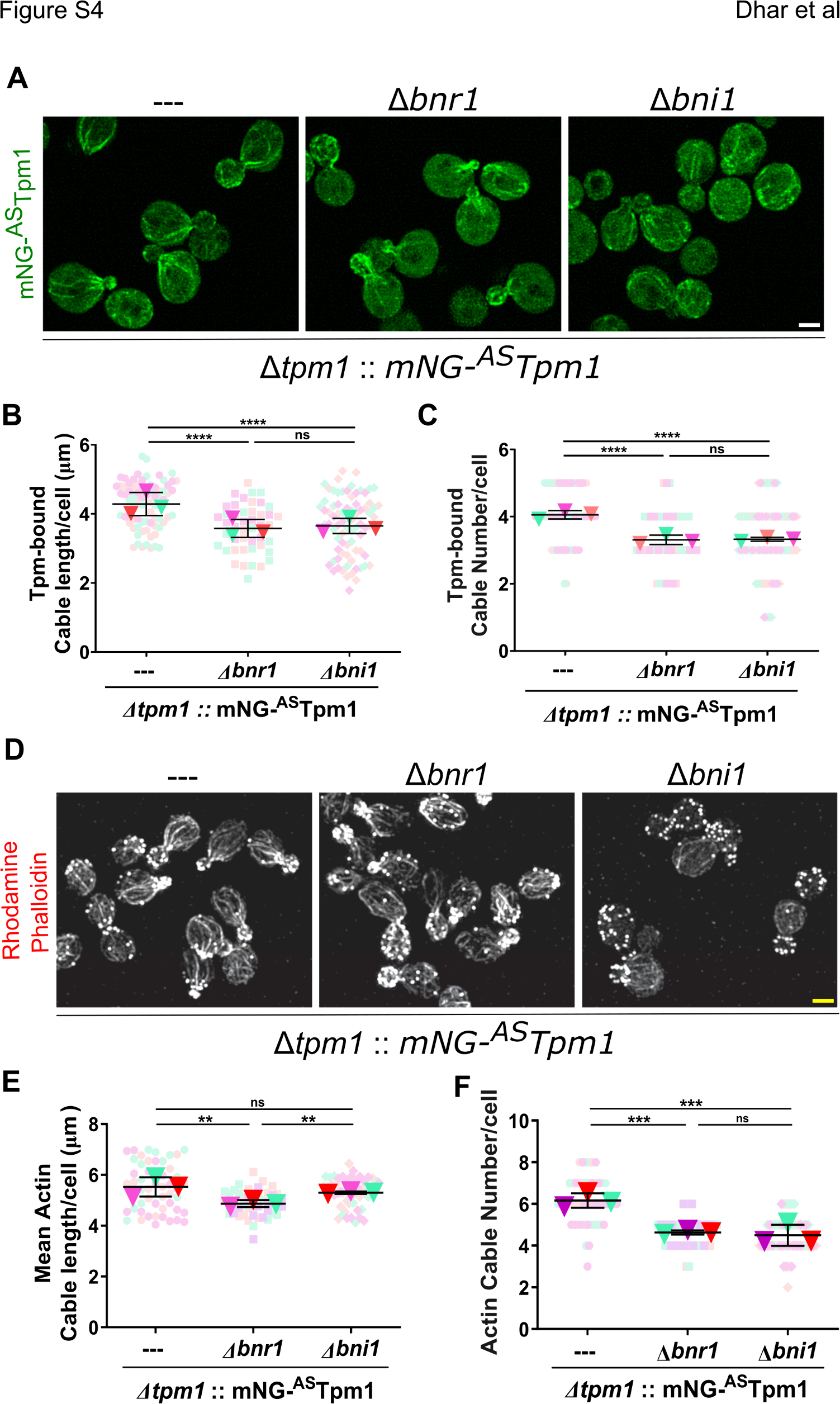

Supplement: S4 Fig — (A) Representative images of Δtpm1 cells expressing mNG-ASTpm1 fusion protein as sole copy with indicated genotypes; scale bar - 2μm. (B) Superplot representing mean Tpm-bound cable length per cell in indicated strains expressing mNG-ASTpm1 as sole copy; n > 15 cells per strain per replicate, N = 3. (C) Superplot representing mean Tpm-bound cable number per cell in indicated strains expressing mNG-ASTpm1 as a sole copy; n ≥ 15 cells per strain per replicate, N = 3. (D) Representative images of Δtpm1 cells expressing mNG-ASTpm1 fusion protein as sole copy with indicated genotypes stained with Rhodamine-phalloidin; scale bar - 2μm. (E) Superplot representing mean actin cable length per cell in indicated yeast strains shown in (D); n = 15 cells per strain per replicate, N = 3. (F) Superplot representing mean actin cable number per cell in in indicated yeast strains shown in (D); n = 15 per strain per replicate, N = 3. (Superplots represent datapoints and means from three independent biological replicates marked in different colours; Kruskal-Wallis test with Dunn’s Multiple Comparisons test was used in (B), (C), (E), (F); * p < 0.05, ** p < 0.01, *** p < 0.001, **** p < 0.0001) (TIF) [file pgen.1011859.s004.tif]

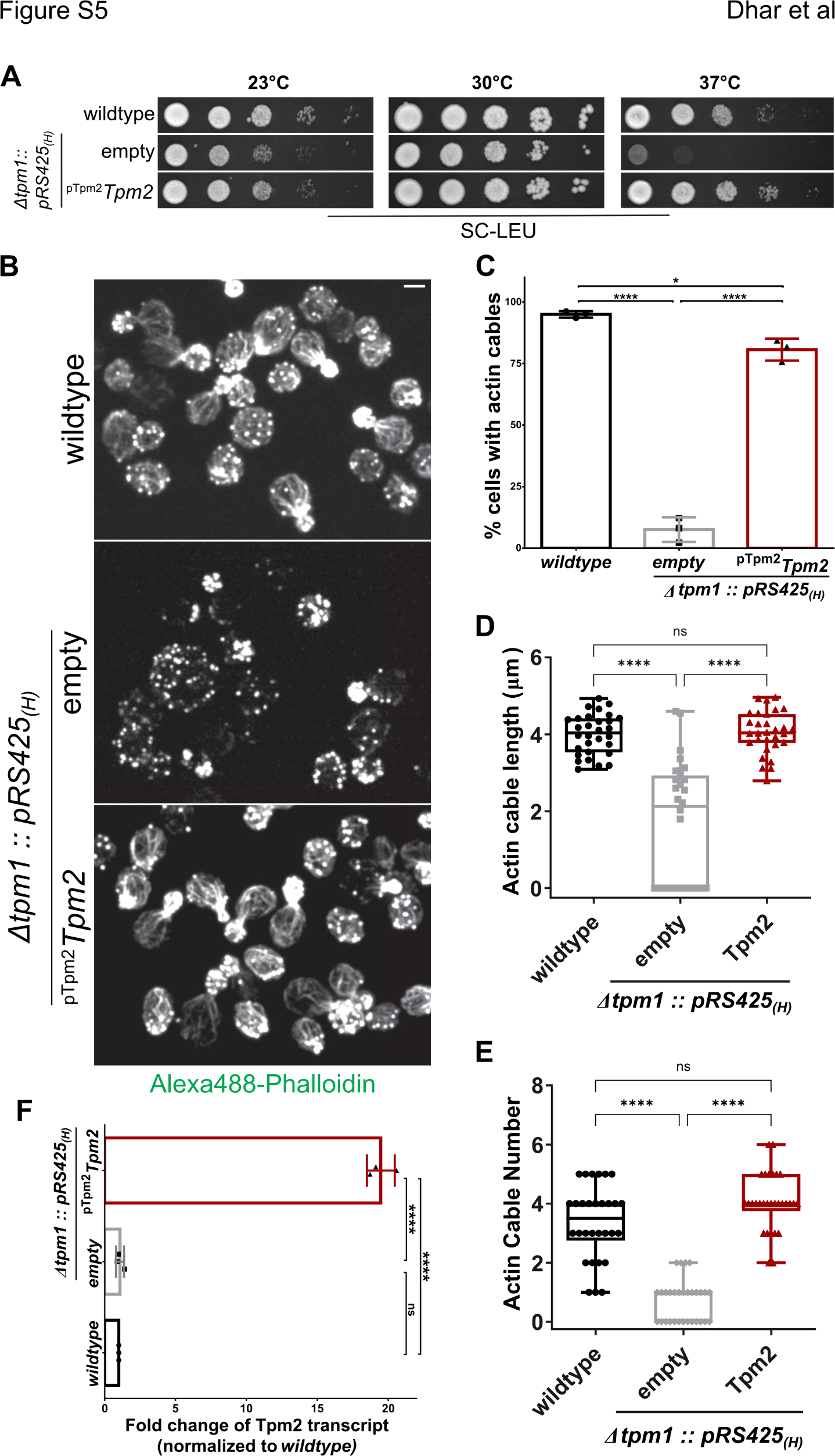

Supplement: S5 Fig — (A) Spot assay image for indicated yeast strains performed at 23°C, 30°C, and 37°C. (B) Representative images of indicated yeast strains stained with Alexa488-phalloidin; scale bar – 2μm. (C) Plot representing mean percentage of cells with detectable actin cables in indicated yeast strains averaged over 3 biological replicates; n ≥ 200 cells for each strain per replicate, N = 3. (D) Plot representing mean actin cable length per cell in the indicated yeast strains as per experiment in (A); n = 30 cells per strain. (E) Plot representing actin cable number per cell in the indicated yeast strains as per experiment in (A); n = 30 cells for each strain. (F) Plot representing fold change of Tpm2 transcript levels normalized to wildtype in the indicated yeast strains containing high-copy number plasmids; n = 3 per strain per experiment, N = 3. (Box represents 25th and 75th percentile, line represents median, whiskers represent minimum and maximum value; One-Way Anova with Tukey’s Multiple Comparisons test was used in (C), (D) and (E). * p < 0.05, ** p < 0.01, *** p < 0.001, **** p < 0.0001) (TIF) [file pgen.1011859.s005.tif]

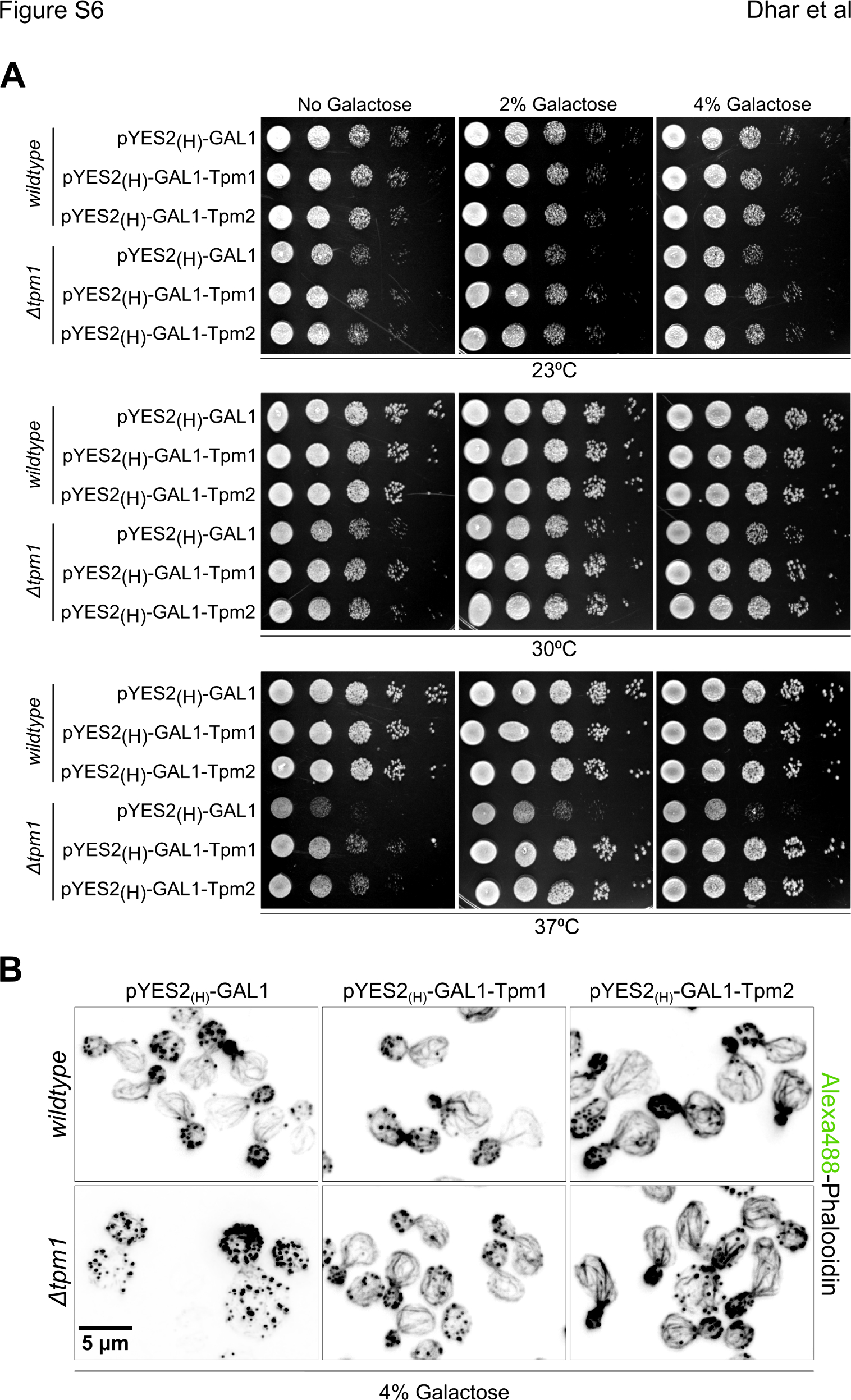

Supplement: S6 Fig — (A) Spot assay image for indicated yeast strains performed at 23°C, 30°C, and 37°C in the presence of 0%, 2%, and 4% galactose. (B) Representative images of cells with indicated genotypes grown in 4% galactose overnight and stained with Alexa488-Phalloidin. (TIF) [file pgen.1011859.s006.tif]

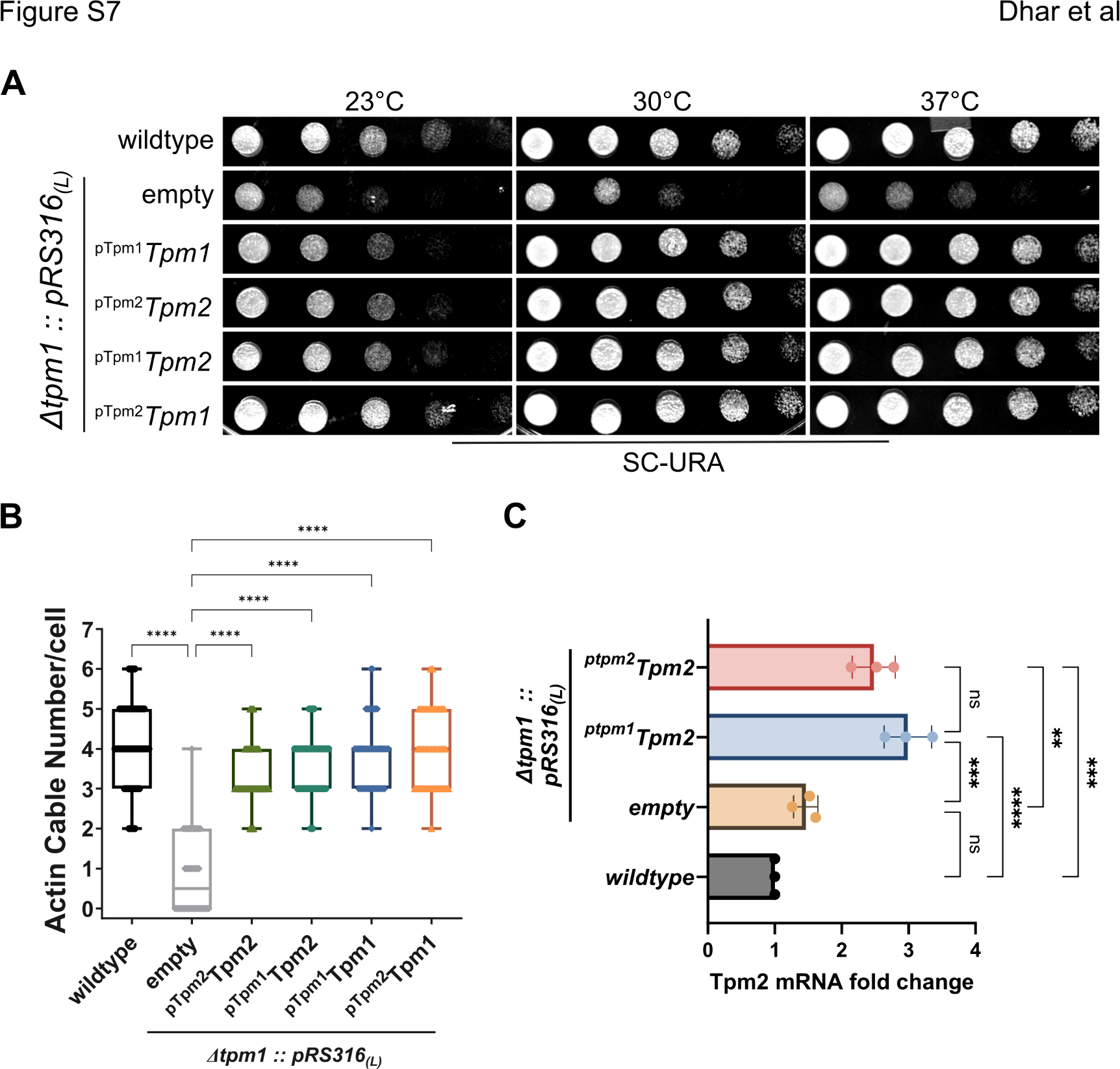

Supplement: S7 Fig — (A) Spot assay image for indicated yeast strains performed at 23°C, 30°C, and 37°C. (B) Plot representing actin cable number per cell in the indicated yeast strains as per experiment in Fig 5A; n = 50 cells per strain. (C) Plot representing fold change of Tpm2 transcript levels normalized to wildtype in the indicated yeast strains containing low-copy number centromeric plasmids; n = 3 per strain per experiment, N = 3. (TIF) [file pgen.1011859.s007.tif]

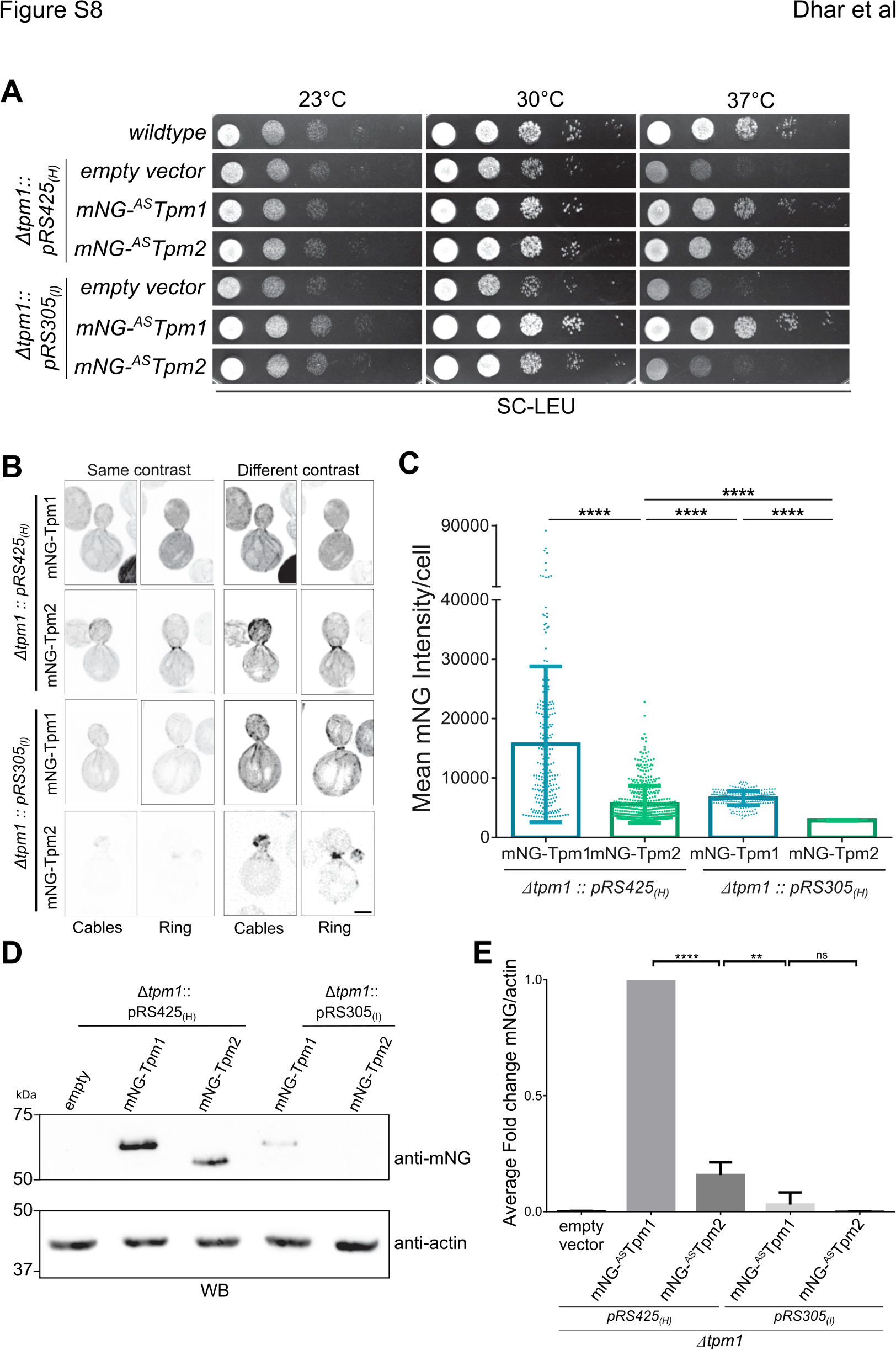

Supplement: S8 Fig — (A) Spot Assay showing growth of indicated yeast strains at 23°C, 30°C, and 37°C. (B) Representative images of yeast cells with indicated genotypes, scale bar - 2μm. (C) Plot showing mean mNG fluorescence intensity per cell in the indicated yeast strains, n > 174 cells per strain. (D) Representative image of western blot probed with anti-mNG and anti-actin (loading control). (E) Plot showing normalized fold change of mNG/actin signal intensity for blot shown in (D), N = 3. (One-Way Anova with Tukey’s Multiple Comparisons test was used in (C) and (E). * p < 0.05, ** p < 0.01, *** p < 0.001, **** p < 0.0001) (TIF) [file pgen.1011859.s008.tif]

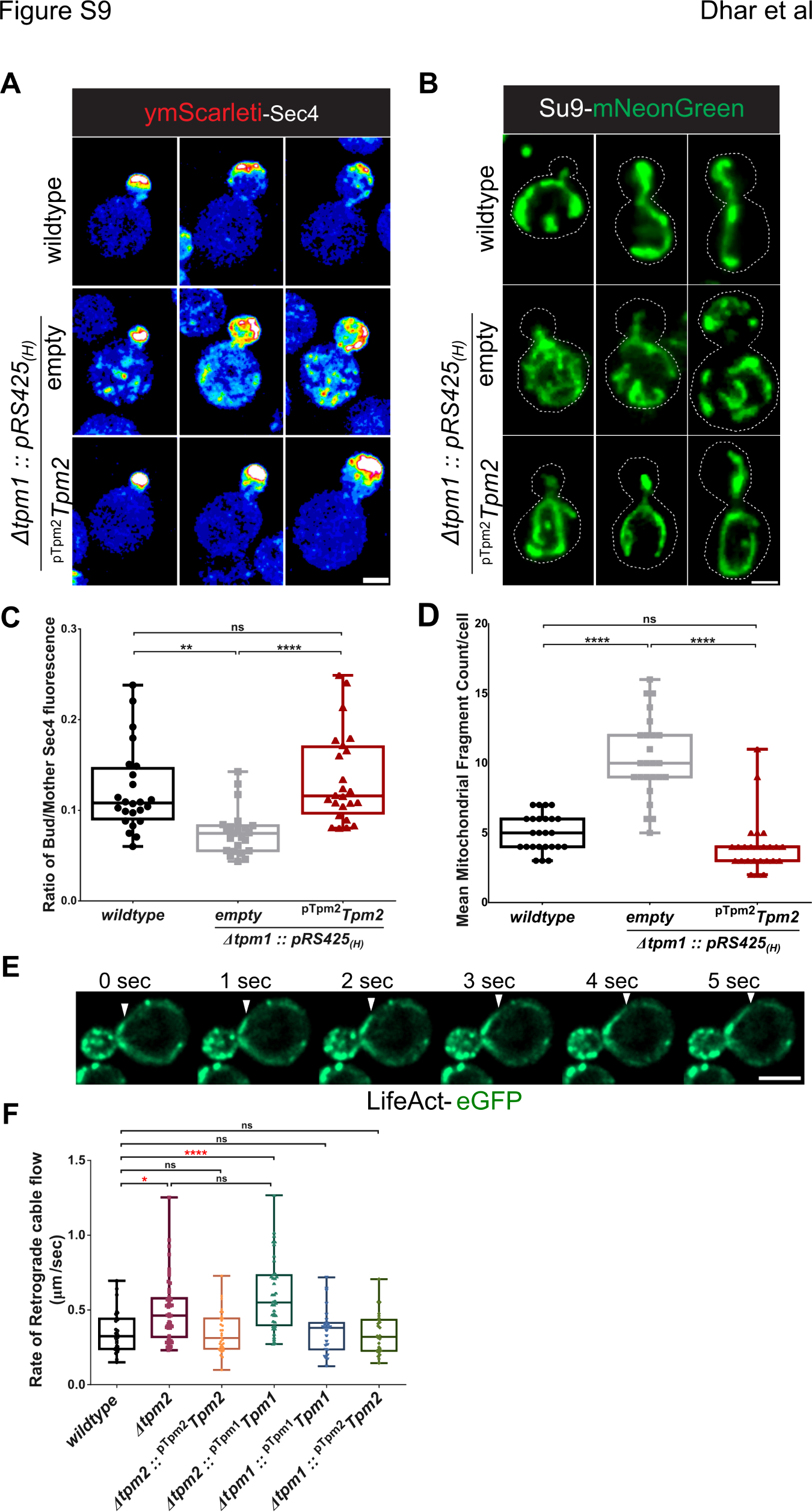

Supplement: S9 Fig — (A) Representative images of indicated yeast strains expressing ymScarleti-Sec4; scale bar – 2μm. (B) Representative images of indicated yeast strains expressing Su9-mNeonGreen; scale bar – 2μm. (C) Plot representing ratio of bud/mother ymScarleti-Sec4 fluorescence per cell in the indicated yeast strains; n ≥ 24 cells per strain. (D) Plot representing mean mitochondrial fragment count per cell in the indicated yeast strains; n ≥ 25 cells per strain. (E) Representative time-lapse montages of wildtype yeast cells expressing LifeAct-eGFP from its native locus, scale bar - 2μm. (F) Plot representing retrograde actin cable flow rate (μm/s) in the indicated yeast strains; n ≥ 20 events per strain. (Box represents 25th and 75th percentile, line represents median, whiskers represent minimum and maximum value; One-Way Anova with Tukey’s Multiple Comparisons test was used in (C), (F); Kruskal-Wallis test with Dunn’s multiple comparisons was used in (D); * p < 0.05, ** p < 0.01, *** p < 0.001, **** p < 0.0001) (TIF) [file pgen.1011859.s009.tif]

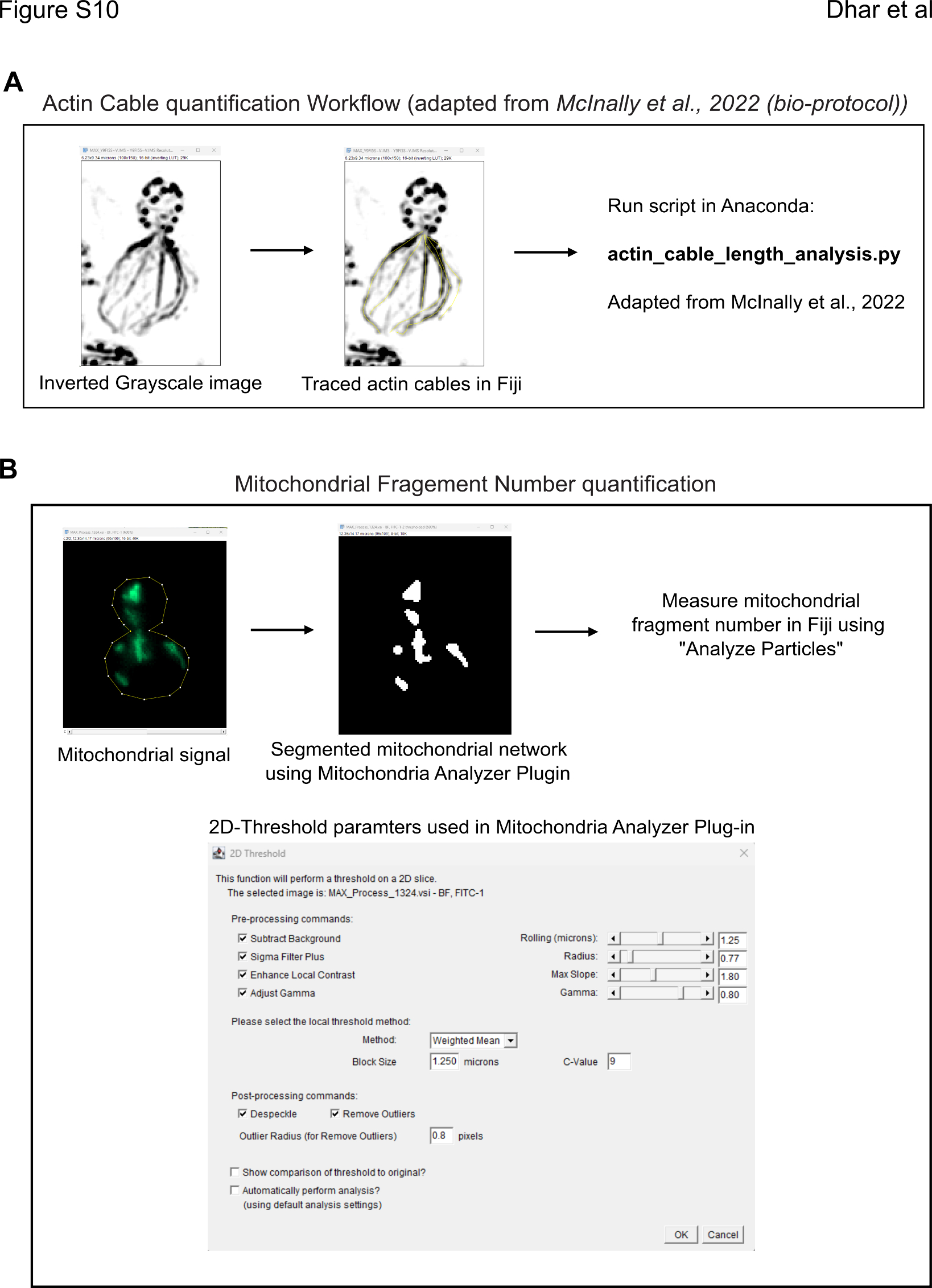

Supplement: S10 Fig — Analysis pipelines used for quantitative analysis of actin cables and mitochondrial morphology in our study. (A) Schematic showing quantification workflow for actin cables as adapted from McInally et al. 2022 (bio-protocol) [120]. (B) Schematic showing quantification workflow for mitochondrial morphology analysis using Mitochondria Analyzer Plug-In (https://github.com/AhsenChaudhry/Mitochondria-Analyzer) in Fiji [124]. (TIF) [file pgen.1011859.s010.tif]
