# Supplementary material for: Functional redundancy and formin-isoform independent localization of tropomyosin paralogs in Saccharomyces cerevisiae
Source: PLoS Genet. 2025 Sep 9;21(9):e1011859. doi: 10.1371/journal.pgen.1011859 (PMC12440208; doi:10.1371/journal.pgen.1011859)
Supplement: S1 Table — List of plasmids used in this study. (DOCX) [file pgen.1011859.s015.docx]

**S1 Table. List of plasmids used in this study.**

| **Plasmid Number** | **Description** | **Source** |
| --- | --- | --- |
| piSP1 | *pRS305_(I)_* | *Sikorski and Hieter, 1989 [1]* |
| piSP5 | *pRS315_(L)_* | *Sikorski and Hieter, 1989 [1]* |
| piSP6 | *pRS316_(L)_* | *Sikorski and Hieter, 1989 [1]* |
| piSP11 | *pRS425_(H)_* | *Christianson et al. 1992 [2]* |
| piSP14 | *pYM25 (yeGFP-hphNT2)* | *Janke et al. 2004 [3]* |
| piSP346 | *pRS305_(I)_-pTpm1-mNG-40L-Tpm1-tCYC* | This Study |
| piSP348 | *pRS305_(I)_-pTpm2-mNG-40L-Tpm2-tCYC* | This Study |
| piSP1422 | *pRS305_(I)_-pTpm1-mNG-40L-^AS^Tpm1-tTpm1* | This Study |
| piSP1423 | *pRS305_(I)_-pTpm2-mNG-40L-^AS^Tpm2-tTpm2* | This Study |
| piSP1430 | *pRS316_(L)_-pTpm1-Tpm1-tTpm1* | This Study |
| piSP1493 | *pRS425_(H)_-pTpm2-Tpm2-tTpm2* | This Study |
| piSP1450 | *pRS316_(L)_-pTpm1-Tpm2-tTpm2* | This Study |
| piSP1451 | *pRS306_(I)_-pADH-ymScarlet-Sec4* | This Study |
| piSP1529 | *pRS316_(L)_-pTpm2-Tpm2-tTpm2* | This Study |
| piSP1534 | *pET28a-6His-Cof1* | This Study |
| piSP1541 | *pRS316_(L)_-pTpm2-Tpm1-tTpm1* | This Study |
| piSP1644 | *pETMCN-^AS^Tpm1* | This Study |
| piSP1646 | *pETMCN-^AS^Tpm2* | This Study |
| piSP1825 | *pRS305_(I)_-pADH-ymScarlet-Sec4-tCYC* | This Study |
| piSP1826 | *pRS315_(L)_-pTpm1-Tpm2-tTpm2* | This Study |
| piSP1827 | *pRS315_(L)_-pTpm2-Tpm1-tTpm1* | This Study |
| piSP1828 | *pRS315_(L)_-pTpm2-Tpm2-tTpm2* | This Study |
| piSP1829 | *pRS305_(I)_-pCYC-Su9-mNG-tCYC* | This Study |
| piSP1830 | *pRS306_(I)_-pCYC-Su9-mNG-tCYC* | This Study |
| piSP1831 | *pRS315_(L)_-pTpm1-Tpm1-tTpm1* | This Study |
| piSP1836 | *pRS306_(I)_-pTpm1-ymScarleti-40L-^AS^Tpm1-tTpm1* | This Study |
| piSP2034 | *pRS305_(I)_-pADH-LifeAct-eGFP-tCYC* | This Study |
| piSP2117 | *pYES2_(H)_-GAL1-Tpm1-tCYC* | This Study |
| piSP2118 | *pYES2_(H)_-GAL1-Tpm2-tCYC* | This Study |

**References**

1. Sikorski RS, Hieter P. A system of shuttle vectors and yeast host strains designed for efficient manipulation of DNA in Saccharomyces cerevisiae. Genetics. 1989;122: 19–27. doi:10.1093/genetics/122.1.19

2. Christianson TW, Sikorski RS, Dante M, Shero JH, Hieter P. Multifunctional yeast high-copy-number shuttle vectors. Gene. 1992;110: 119–122. doi:10.1016/0378-1119(92)90454-w

3. Janke C, Magiera MM, Rathfelder N, Taxis C, Reber S, Maekawa H, et al. A versatile toolbox for PCR-based tagging of yeast genes: new fluorescent proteins, more markers and promoter substitution cassettes. Yeast. 2004;21: 947–962. doi:10.1002/yea.1142
