# Supplementary material for: Functional redundancy and formin-isoform independent localization of tropomyosin paralogs in Saccharomyces cerevisiae
Source: PLoS Genet. 2025 Sep 9;21(9):e1011859. doi: 10.1371/journal.pgen.1011859 (PMC12440208; doi:10.1371/journal.pgen.1011859)
Supplement: S2 Table — List of yeast strains used in this study. (DOCX) [file pgen.1011859.s016.docx]

**S2 Table. List of yeast strains used in this study.**

| **Strain Number** | **Genotype** | **Source** |
| --- | --- | --- |
| YSP002 | *MATa ura3-52 leu2∆1 trp1∆63 his3∆200* | *Pereira and Schiebel*, 2001 [1] |
| YSP003 | *MATα ura3-52 lys2-801amber ade2-101ochre trp1∆63 his3∆200 leu2∆1* | *Sikorski and Hieter*, 1989 [2] |
| YSP107 | *MATa ura3-52 leu2∆1 trp1∆63 his3∆200 pRS305_(I)_-pTpm1-mNG-40aaL-Tpm1-tTpm1* | This study |
| YSP108 | *MATa ura3-52 leu2∆1 trp1∆63 his3∆200 pRS305_(I)_-pTpm2-mNG-40aaL-Tpm2-tTpm2* | This study |
| YSP146 | *MATa ura3-52 leu2∆1 trp1∆63 his3∆200 -∆tpm1-HIS3MX6-pRS305_(I)_-pTpm1-mNG-40L-Tpm1-tTpm1* | This study |
| YSP195 | *MATa ura3-52 leu2∆1 trp1∆63 his3∆200-∆tpm1-HIS3MX6* | This study |
| YSP196 | *MATa ura3-52 leu2∆1 trp1∆63 his3∆200-∆tpm2-HIS3MX6* | This study |
| YSP227 | *MATa ura3-52 leu2∆1 trp1∆63 his3∆200 -∆tpm1-HIS3MX6-pRS305_(I)_* | This study |
| YSP355 | *MATa ura3-52 leu2∆1 trp1∆63 his3∆200 -pRS305_(I)_-pTpm1-mNG-40aaL-^AS^Tpm1-tTpm1* | This study |
| YSP356 | *MATa ura3-52 leu2∆1 trp1∆63 his3∆200 -pRS305_(I)_-pTpm2-mNG-40aaL-^AS^Tpm2-tTpm2* | This study |
| YSP419 | *MATa ura3-52 leu2∆1 trp1∆63 his3∆200 -∆tpm1-HIS3MX6-pRS305_(I)_-pTpm1-mNG-40L-^AS^Tpm1-tTpm1* | This study |
| YSP440 | *MATa ura3-52 leu2∆1 trp1∆63 his3∆200 -pRS305_(I)_-pTpm1-mNG-40aaL-^AS^Tpm1-tTpm1 -∆bnr1-HIS3MX6* | This study |
| YSP441 | *MATa ura3-52 leu2∆1 trp1∆63 his3∆200 -pRS305_(I)_-pTpm2-mNG-40aaL-^AS^Tpm2-tTpm2-∆bnr1-HIS3MX6* | This study |
| YSP444 | *MATa ura3-52 leu2∆1 trp1∆63 his3∆200 -pRS305_(I)_-pTpm2-mNG-40aaL-^AS^Tpm2-tTpm2-∆bni1-HIS3MX6* | This study |
| YSP443 | *MATa ura3-52 leu2∆1 trp1∆63 his3∆200 -pRS305_(I)_-pTpm1-mNG-40aaL-^AS^Tpm1-tTpm1 -∆bni1-HIS3MX6* | This study |
| YSP609 | *MATa ura3-52 leu2∆1 trp1∆63 his3∆200 -∆tpm1-HIS3MX6-pRS305_(I)_-pTpm1-mNG-40L-Tpm1-tTpm1 - OM45-3XmCherry-Kan* | This study |
| YSP610 | *MATa ura3-52 leu2∆1 trp1∆63 his3∆200 -∆tpm1-HIS3MX6-OM45-3XmCherry-Kan* | This study |
| YSP611 | *MATa ura3-52 leu2∆1 trp1∆63 his3∆200 -∆tpm1-HIS3MX6-pRS305_(I)_-pTpm1-mNG-40L-^AS^Tpm1-tTpm1 -OM45-3XmCherry-Kan* | This study |
| YSP612 | *MATa ura3-52 leu2∆1 trp1∆63 his3∆200 --OM45-3XmCherry-Kan* | This study |
| YSP671 | *MATa ura3-52 leu2∆1 trp1∆63 his3∆200 -pRS305_(I)_-pTpm2-mNG-40aaL-^AS^Tpm2-tTpm2 - pRS306_(I)_-pTpm1-ymScarletiI-40aaLinker-^AS^Tpm1-tTpm1* | This study |
| YSP685 | *MATa ura3-52 leu2∆1 trp1∆63 his3∆200 - ∆tpm1-HIS3MX6-pRS425_(H)_* | This study |
| YSP686 | *MATa ura3-52 leu2∆1 trp1∆63 his3∆200 -∆tpm1-HIS3MX6-pRS425_(H)_-pTpm2-Tpm2-tTpm2* | This study |
| YSP767 | *MATa ura3-52 leu2∆1 trp1∆63 his3∆200 -∆tpm1-HIS3MX6 - pRS425_(H)_-pTpm2-mNG-40L-^AS^Tpm2-tTpm2* | This study |
| YSP780 | *MATa ura3-52 leu2∆1 trp1∆63 his3∆200 -∆tpm1-his3MX6-pRS305_(I)_-pTpm1-mNG-40L-^AS^Tpm1-tTpm1 - ∆bnr1-hphNT1* | This study |
| YSP794 | *MATa ura3-52 leu2∆1 trp1∆63 his3∆200 -∆tpm1-HIS3MX6 - pRS425_(H)_-pTpm1-mNG-40L-^AS^Tpm1-tTpm1* | This study |
| YSP824 | *MATa ura3-52 leu2∆1 trp1∆63 his3∆200 -∆tpm1-HIS3MX6 - pRS306_(I)_-pADH-ymScarleti-Sec4* | This study |
| YSP835 | *MATa ura3-52 leu2∆1 trp1∆63 his3∆200 - pRS425_(H)_ - pRS316_(L)_* | This study |
| YSP836 | *MATa ura3-52 leu2∆1 trp1∆63 his3∆200 - pRS306_(I)_-pADH-ymScarleti-Sec4* | This study |
| YSP837 | *MATa ura3-52 leu2∆1 trp1∆63 his3∆200 -∆tpm1-HIS3MX6-pRS425_(H)_-pTpm2-Tpm2-tTpm2 - pRS306_(I)_-pADH-ymScarleti-Sec4* | This study |
| YSP848 | *MATa ura3-52 leu2∆1 trp1∆63 his3∆200 -∆tpm1-HIS3MX6 - pRS316_(L)_-pTpm2-Tpm1-tTpm1* | This study |
| YSP887 | *MATa ura3-52 leu2∆1 trp1∆63 his3∆200 -∆tpm1-HIS3MX6 - pRS316_(L)_* | This study |
| YSP907 | *MATa ura3-52 leu2∆1 trp1∆63 his3∆200∆tpm1-HIS3MX6; pRS316_(L)_-pTpm1-Tpm1-tTpm1* | This study |
| YSP909 | *MATa ura3-52 leu2∆1 trp1∆63 his3∆200∆tpm1-HIS3MX6; pRS316_(L)_-pTpm2-Tpm2-tTpm2* | This study |
| YSP1090 | *MATa ura3-52 leu2∆1 trp1∆63 his3∆200-∆tpm1-HIS3MX6; pRS316_(L)_-pTpm1-Tpm2-tTpm2* | This study |
| YSP1117 | *MATa ura3-52 leu2∆1 trp1∆63 his3∆200-pRS306_(I)_-pCYC-Su9-mNG-tCYC - pRS425_(H)_* | This study |
| YSP1118 | *MATa ura3-52 leu2∆1 trp1∆63 his3∆200-∆tpm1-HIS3MX6-pRS425_(H)_ - pRS306_(I)_-pCYC-Su9-mNG-tCYC* | This study |
| YSP1119 | *MATa ura3-52 leu2∆1 trp1∆63 his3∆200-∆tpm1-HIS3MX6-pRS425_(H)_-pTpm2-Tpm2-tTpm2 - pRS306_(I)_-pCYC-Su9-mNG-tCYC* | This study |
| YSP1120 | *MATa ura3-52 leu2∆1 trp1∆63 his3∆200-∆tpm1-HIS3MX6 - pRS306_(I)_-pADH-ymScarleti-Sec4 - pRS315_(L)_* | This study |
| YSP1121 | *MATa ura3-52 leu2∆1 trp1∆63 his3∆200-∆tpm1-HIS3MX6 - pRS306_(I)_-pADH-ymScarleti-Sec4 - pRS315_(L)_-pTpm1-Tpm2-tTpm2* | This study |
| YSP1122 | *MATa ura3-52 leu2∆1 trp1∆63 his3∆200-∆tpm1-HIS3MX6 - pRS306_(I)_-pADH-ymScarleti-Sec4 - pRS315_(L)_-pTpm2-Tpm1-tTpm1* | This study |
| YSP1123 | *MATa ura3-52 leu2∆1 trp1∆63 his3∆200-∆tpm1-HIS3MX6 - pRS306_(I)_-pADH-ymScarleti-Sec4 - pRS315_(L)_-pTpm2-Tpm2-tTpm2* | This study |
| YSP1126 | *MATa ura3-52 leu2∆1 trp1∆63 his3∆200 pRS305_(I)_-pCYC-Su9-mNG-tCYC pRS316* | This study |
| YSP1127 | *MATa ura3-52 leu2∆1 trp1∆63 his3∆200 ∆tpm1-HIS3MX6 -pRS305_(I)_-pCYC-Su9-mNG-tCYC* | This study |
| YSP1128 | *MATa ura3-52 leu2∆1 trp1∆63 his3∆200 ∆tpm1-HIS3MX6 - pRS316_(L)_-pTpm2-Tpm1-tTpm1 - pRS305_(I)_-pCYC-Su9-mNG-tCYC* | This study |
| YSP1129 | *MATa ura3-52 leu2∆1 trp1∆63 his3∆200 ∆tpm1-HIS3MX6; pRS316_(L)_-pTpm1-Tpm1-tTpm1- pRS305_(I)_-pCYC-Su9-mNG-tCYC* | This study |
| YSP1130 | *MATa ura3-52 leu2∆1 trp1∆63 his3∆200 ∆tpm1-HIS3MX6; pRS316_(L)_-pTpm2-Tpm2-tTpm2- pRS305_(I)_-pCYC-Su9-mNG-tCYC* | This study |
| YSP1131 | *MATa ura3-52 leu2∆1 trp1∆63 his3∆200 ∆tpm1-HIS3MX6; pRS316_(L)_-pTpm1-Tpm2-tTpm2- pRS305_(I)_-pCYC-Su9-mNG-tCYC* | This study |
| YSP1150 | *MATa ura3-52 leu2∆1 trp1∆63 his3∆200-∆tpm1-HIS3MX6 - pRS306_(I)_-pADH-ymScarleti-Sec4 , pRS315_(L)_-pTpm1-Tpm1-tTpm1* | This study |
| YSP1189 | *MATa ura3-52 leu2∆1 trp1∆63 his3∆200-Abp140-GFP-KanMX6* | This study |
| YSP1191 | *MATa ura3-52 leu2∆1 trp1∆63 his3∆200-∆tpm2-HIS3MX6 - Abp140-GFP-KanMX6* | This study |
| YSP1192 | *MATa ura3-52 leu2∆1 trp1∆63 his3∆200-∆tpm2-HIS3MX6) - pRS425_(H)_-pTpm2-Tpm2-tTpm2) - Abp140-GFP-KanMX6* | This study |
| YSP1577 | *MATa ura3-52 leu2∆1 trp1∆63 his3∆200 ∆tpm1-His3MX6-∆bni1-hph - pRS305_(I)_-pTpm1-mNG-40L-^AS^Tpm1-tTpm* | This study |
| YSP1639 | *MATa ura3-52 leu2∆1 trp1∆63 his3∆200 - ∆tpm1-his3MX6 - pRS305_(I)_-pTpm2-mNG-^AS^Tpm2-tTpm2* | This study |
| YSP1816 | *MATa ura3-52 leu2∆1 trp1∆63 his3∆200 pRS305_(I)_-pADH-LifeAct-eGFP-tCYC - ∆tpm1::his3MX6 - pRS316_(L)_ (empty)* | This study |
| YSP1817 | *MATa ura3-52 leu2∆1 trp1∆63 his3∆200 pRS305_(I)_-pADH-LifeAct-eGFP-tCYC - ∆tpm2::his3MX6 - pRS316_(L)_ (empty)* | This study |
| YSP1818 | *MATa ura3-52 leu2∆1 trp1∆63 his3∆200 pRS305_(I)_-pADH-LifeAct-eGFP-tCYC - pRS316_(L)_ (empty)* | This study |
| YSP1819 | *MATa ura3-52 leu2∆1 trp1∆63 his3∆200 pRS305_(I)_-pADH-LifeAct-eGFP-tCYC - ∆tpm1::his3MX6 - pRS316_(L)_-pTpm1-Tpm1-tTpm1* | This study |
| YSP1820 | *MATa ura3-52 leu2∆1 trp1∆63 his3∆200 pRS305_(I)_-pADH-LifeAct-eGFP-tCYC - ∆tpm2::his3MX6 - pRS316_(L)_-pTpm1-Tpm1-tTpm1* | This study |
| YSP1822 | *MATa ura3-52 leu2∆1 trp1∆63 his3∆200 pRS305_(I)_-pADH-LifeAct-eGFP-tCYC - ∆tpm1::his3MX6 - pRS316_(L)_-pTpm1-Tpm1-tTpm1* | This study |
| YSP1823 | *MATa ura3-52 leu2∆1 trp1∆63 his3∆200 pRS305_(I)_-pADH-LifeAct-eGFP-tCYC - ∆tpm2::his3MX6 - pRS316_(L)_-pTpm1-Tpm1-tTpm1* | This study |

**References**

1. Pereira G, Schiebel E. The role of the yeast spindle pole body and the mammalian centrosome in regulating late mitotic events. Current Opinion in Cell Biology. 2001;13: 762–769. doi:10.1016/S0955-0674(00)00281-7

2. Sikorski RS, Hieter P. A system of shuttle vectors and yeast host strains designed for efficient manipulation of DNA in Saccharomyces cerevisiae. Genetics. 1989;122: 19–27. doi:10.1093/genetics/122.1.19
