# Supplementary material for: Functional redundancy and formin-isoform independent localization of tropomyosin paralogs in Saccharomyces cerevisiae
Source: PLoS Genet. 2025 Sep 9;21(9):e1011859. doi: 10.1371/journal.pgen.1011859 (PMC12440208; doi:10.1371/journal.pgen.1011859)
Supplement: Attachment — Submitted filename: Reviewers comments_FINAL.docx [file pgen.1011859.s019.docx]

**Reviewer Comments for Manuscript entitled “**Functional redundancy and formin-independent localization of tropomyosin isoforms in Saccharomyces cerevisiae”

The manuscript by Dhar *et al.* investigates the differences between the two budding yeast tropomyosin homologs, Tpm1 and Tpm2. The authors utilize a recently published fluorescently tagged version of Tpm1 and Tpm2 to explore their localization and functionality. This work is relevant to the field as it contributes to our understanding of the specific roles of tropomyosin homologs in budding yeast cells and may shed some light on the multiple isoforms found in higher eukaryotes. The *in vitro* binding assays, which demonstrate comparable protection of actin filaments against cofilin severing by purified Tpm1 and Tpm2, suggest a degree of functional redundancy between the two homologs.

While the research presented holds potential merit, I found certain aspects of the manuscript challenging to understand, which hindered a full appreciation of the work. To enhance clarity and ensure the work is accessible to a broader readership, I recommend addressing the following points:

**Experimental Design and Justification:** It would be beneficial to include a more explicit rationale for the design of specific experiments. Clearly articulating the reasoning behind each experimental approach would strengthen the overall logical flow of the manuscript.

**Genetic Background Details:** Related to the above point, providing a more thorough justification for the specific genetic backgrounds used in the study would help readers understand their relevance to the experimental questions.

**Methodological Context:** Given the reliance on a recently published method for tagging tropomyosins, a brief background or more detailed description of this method would be helpful for readers who may not be familiar with it in the introduction. This would allow for a more comprehensive understanding of the experimental setup.

**Clarity and Precision in Terminology:** There were several instances of typos and references to the wrong figures. Fixing these mistakes would significantly enhance readability and prevent potential misinterpretations. A careful review of all figures and text for accuracy is necessary.

In summary, a focused revision on the clarity and logical presentation of the writing would greatly improve the manuscript's accessibility, allowing both expert and less expert readers to fully understand the significance of the work and critically evaluate the results.

Below, I provide examples of specific sections that could benefit from revision. Please note that this list is illustrative, and a comprehensive review of the entire manuscript for clarity and justification is recommended.

**Major concerns:**

1- The title does not represent the findings. It is inaccurate to write “formin-independent” as Tpm1 and Tpm2 bind actin cables nucleated by formins Bnr1 and Bni1. You mean to say that the “localization of Tpm1 and Tpm2 is agnostic of formin type”. Please revise the title for clarity.

2- The introduction lacks relevant details about previous experiments that tested the localization and functionality of Tromomyosins. This background information is critical to understand how your approach is different and why it may successfully demonstrate homolog specific functions of tropomyosins. For example, what’s the main difference that makes these fluorescently tagged tropomyosins functional? How was functionality tested before? Growth assays, quantitative imaging of the actin cytoskeleton, functional assays of the actin cables…?

3- The authors emphasize the improved capability of the”AS” versions of Tpm1 and Tpm2 to rescue the length and number of actin cables of *∆tmp1* mutant cells over the non-AS versions with data from Figure 1 supporting this difference. However, the data in figures S1C, D-F and I show no differences between the AS and non-AS versions in rescuing growth, actin cables and mitochondria distribution. The discussion doesn’t address this discrepancy. A simple explanation acknowledging that a full complement of full-length actin cables appears to not be necessary for function.

4- The authors hypothesize that Tpm1 and Tpm2 do not display a bias for actin filaments nucleated by Bnr1 or Bni1. Although I don’t believe that Tpm1 and Tpm2 only bind to either Bni1 or Bnr1 actin cable networks based on the protein distributions shown in Figure 1A and B, the data used to support this lack of bias is not convincing.

First, to test this hypothesis, the authors visualize the localization of mNG-AS-Tpm1 and mNG-AS-Tpm2 in *∆bnr1* and in *∆bni1* cells. The rationale for using this approach to test their hypothesis is missing from the results. It is also impossible to discern the actin decorated cables above the green background in figures 2A and D. Using a black and white inverted lookup table may help. Finally, the authors overlook the possibility that removing one of the two main actin cable networks with the deletion of the formin responsible for their nucleation may alter the distribution of the Tpms. This possible interpretation is missing from the discussion.

Second, co-expression of ymScarleti-AS-Tpm1 and mNG-AS-Tpm2 was used to show colocalization of both tropomyosins to the same actin cable network. However, figure 2G shows little colocalization of the two tropomyosins. Indeed, there is more distinct localization (green and purple signals) than co-localization (white signal) between the two proteins.

Finally, the data shown in figures 1A and B show the localization of each Tpm1 and Tpm2 constructs to both Bni1 and Bnr1 networks and could be used to support the lack of bias of each tropomyosin for a specific actin cable network. Some enlarged panels with arrows labeling the important actin networks may help illustrate the lack of bias.

5- The authors propose that the low endogenous expression level of Tpm2 is responsible for its inability to rescue *∆tpm1* phenotypes. This is based on the in vitro experiments that show that purified Tpm1 and Tpm2 can both protect actin filaments from cofilin mediated severing. They show in Figure S4 that overexpression of Tpm2 can indeed rescue the presence of actin cables and the viability of *∆tpm1* cells. The authors claim that actin cable number and length are rescued but no data supports these quantitative measurements.

In figure S4E, how can the fold change of Tpm2 transcript be the same as wildtype when the endogenous transcript is present in addition to the exogenous ^pTpm2^TPM2 expressed from a plasmid?

Finally, the authors mention that even a mild overexpression is sufficient to rescue the phenotypes. Can you clarify what is the minimal amount of Tpm2 you found is sufficient to rescue the *∆tpm1* mutant phenotypes?

6- Clarify the level of expression for the data shown in Figure 6. Which of these data were acquired in cells with high copy plasmid and which ones from cells with low copy plasmids?

7- I’m curious to know why the mNG-AS-Tpm1 and mNG-AS-Tpm2 constructs were not integrated into the endogenous *tpm1* and *tpm2* loci if they are fully functional. Why were they expressed from plasmids or integrated into the leu2 loci?

**Other comments on the figures:**

Figure 1A and B (and S1A and B and 3A and B). Are these three rows of images a timelapse of the same cell? The specific timestamps on each time point are missing.

Figures S1A and B. The images are too small to discern the fluorescent signal.

Figure S1C. What’s the shaded area around each curve? SEM? This data also lacks statistical test to determine whether there is any significant difference between the curves especially *∆tpm1::mNG-Tpm1* and *∆tpm1::mNG-AS-Tpm1.*

Figure S1. The legends for panels H and I are missing.

Figures 2A and D. It is not possible to discern the actin decorated cables above the green background in these images. Using a black and white inverted lookup table may help.

Figure S2G. The lines are too thick and the markers can’t be distinguished from each other.

Figure S5C and E. Can you provide an explanation for the discrepancy between the trends seen in C versus the trend seen in E.

**Minor comments (this list is not comprehensive):**

1- Line 18. ABP acronym has not been defined. It is defined twice at lines 378 and 440.

2- Line 228. Shouldn’t it say ^pTpm1^TPM2 instead of ^pTpm1^TPM1?

3- Line 279. “We used exogenously expressed…” not “exogenous”.

4- Lines 430 and 431. Refers to figures 4G and H. There are no Figures 4G and H.

5- Line 446. Refers to Figures 5E-G. There are no Figures 5F and G.
