# Supplementary material for: Functional redundancy and formin-isoform independent localization of tropomyosin paralogs in Saccharomyces cerevisiae
Source: PLoS Genet. 2025 Sep 9;21(9):e1011859. doi: 10.1371/journal.pgen.1011859 (PMC12440208; doi:10.1371/journal.pgen.1011859)
Supplement: Attachment — Submitted filename: Response_to_Reviewers-PGENETICS-D-25-00504.docx [file pgen.1011859.s021.docx]

**PGENETICS-D-25-00504**

Functional redundancy and formin-independent localization of tropomyosin isoforms in Saccharomyces cerevisiae: PLOS Genetics

Dear Saravanan,

Thank you for submitting this interesting and exciting manuscript to PLOS Genetics. After careful consideration, we feel that it has merit but does not fully meet PLOS Genetics's publication criteria as it currently stands. Therefore, we invite you to submit a revised version of the manuscript that addresses the points raised during the review process.

The manuscript was reviewed by three reviewers, all of whom are experts in the field.  Two of the reviewers checked the box for minor revisions, and the third reviewer checked the major revision box.  My reading of their reviews is that addressing them will help sharpen the focus and conclusions, and also ensure that the writing is as tight as it can be such that conclusions are fully supported or appropriately nuanced, the figures are referenced appropriately, and the questions raised by review have been addressed and resolved.  The reviewers noted that much of the approach is cell biological, and thus one might see this type of study in MBOC, JCB, MCB.  My own impression is that there is enough of a yeast genetics slant here that PLOS Genetics is just fine from this editor's perspective.  The system automatically provides 60 days for revision. This may be just fine, but if you find you need a bit more time to revise please just let us know.  Please submit your revised manuscript within 60 days Aug 10 2025 11:59PM. If you will need more time than this to complete your revisions, please reply to this message or contact the journal office at [plosgenetics@plos.org](mailto:plosgenetics@plos.org). When you're ready to submit your revision, log on to <https://www.editorialmanager.com/pgenetics/> and select the 'Submissions Needing Revision' folder to locate your manuscript file.  Please include the following items when submitting your revised manuscript:  * A rebuttal letter that responds to each point raised by the editor and reviewer(s). You should upload this letter as a separate file labeled 'Response to Reviewers'. This file does not need to include responses to any formatting updates and technical items listed in the 'Journal Requirements' section below. * A marked-up copy of your manuscript that highlights changes made to the original version. You should upload this as a separate file labeled 'Revised Manuscript with Track Changes'. * An unmarked version of your revised paper without tracked changes. You should upload this as a separate file labeled 'Manuscript'.  If you would like to make changes to your financial disclosure, competing interests statement, or data availability statement, please make these updates within the submission form at the time of resubmission. Guidelines for resubmitting your figure files are available below the reviewer comments at the end of this letter.  We look forward to receiving your revised manuscript and thank you again for submitting this exciting and interesting work to PLOS Genetics.  Kind regards,  Joseph Heitman, MD, PhD Academic Editor PLOS Genetics  Geraldine Butler Section Editor PLOS Genetics  Aimée Dudley Editor-in-Chief PLOS Genetics  Anne Goriely Editor-in-Chief PLOS Genetics

**Journal Requirements:**

1) We ask that a manuscript source file is provided at Revision. Please upload your manuscript file as a .doc, .docx, .rtf or .tex. If you are providing a .tex file, please upload it under the item type u2018LaTeX Source Fileu2019 and leave your .pdf version as the item type u2018Manuscriptu2019.

2) Please provide an Author Summary. This should appear in your manuscript between the Abstract (if applicable) and the Introduction, and should be 150-200 words long. The aim should be to make your findings accessible to a wide audience that includes both scientists and non-scientists. Sample summaries can be found on our website under Submission Guidelines: <https://journals.plos.org/plosgenetics/s/submission-guidelines#loc-parts-of-a-submission>

3) Please upload all main figures as separate Figure files in .tif or .eps format. For more information about how to convert and format your figure files please see our guidelines:  <https://journals.plos.org/plosgenetics/s/figures>

4) We notice that your supplementary Figures, and Tables are included in the manuscript file. Please remove them and upload them with the file type 'Supporting Information'. Please ensure that each Supporting Information file has a legend listed in the manuscript after the references list.

5) Some material included in your submission may be copyrighted. According to PLOSu2019s copyright policy, authors who use figures or other material (e.g., graphics, clipart, maps) from another author or copyright holder must demonstrate or obtain permission to publish this material under the Creative Commons Attribution 4.0 International (CC BY 4.0) License used by PLOS journals. Please closely review the details of PLOSu2019s copyright requirements here: PLOS Licenses and Copyright. If you need to request permissions from a copyright holder, you may use PLOS's Copyright Content Permission form. Please respond directly to this email and provide any known details concerning your material's license terms and permissions required for reuse, even if you have not yet obtained copyright permissions or are unsure of your material's copyright compatibility. Once you have responded and addressed all other outstanding technical requirements, you may resubmit your manuscript within Editorial Manager.  Potential Copyright Issues:

 i) Figures 4D, 7A, and 7B. Please confirm whether you drew the images / clipart within the figure panels by hand. If you did not draw the images, please provide (a) a link to the source of the images or icons and their license / terms of use; or (b) written permission from the copyright holder to publish the images or icons under our CC BY 4.0 license. Alternatively, you may replace the images with open source alternatives. See these open source resources you may use to replace images / clip-art: - [https://commons.wikimedia.org](https://commons.wikimedia.org/) - <https://openclipart.org/>.

The schematic models shown in Fig. 4D, 7A, 7B were drawn in BioRender web application and the publication licenses are attached as a “Related Manuscript file” along with our submission.

ii) Figure S7A appears to have been adapted from a previously published figure. Please provide written permission from the copyright holder to publish this under our CC-BY 4.0 license, or remove the figure / replace the image. Please note we do not recommend using standard request forms available on Publishers' websites, as they grant single use rather than republication under an open access license.

The schematic shown in Fig. S7A was made for this study and not adapted from a previous one. The text in the figure is actually referring to the fact that the quantification pipeline was adopted from the previous study. Hence, no permission should be required for this schematic.

 6) We note that your Data Availability Statement is currently as follows: "All relevant data are within the manuscript and its Supporting information files.". Please confirm at this time whether or not your submission contains all raw data required to replicate the results of your study. Authors must share the “minimal data set” for their submission. PLOS defines the minimal data set to consist of the data required to replicate all study findings reported in the article, as well as related metadata and methods (<https://journals.plos.org/plosone/s/data-availability#loc-minimal-data-set-definition>). For example, authors should submit the following data:

 1) The values behind the means, standard deviations and other measures reported;

 2) The values used to build graphs;

3) The points extracted from images for analysis.. Authors do not need to submit their entire data set if only a portion of the data was used in the reported study. If your submission does not contain these data, please either upload them as Supporting Information files or deposit them to a stable, public repository and provide us with the relevant URLs, DOIs, or accession numbers. For a list of recommended repositories, please see <https://journals.plos.org/plosone/s/recommended-repositories>.  If there are ethical or legal restrictions on sharing a de-identified data set, please explain them in detail (e.g., data contain potentially sensitive information, data are owned by a third-party organization, etc.) and who has imposed them (e.g., an ethics committee). Please also provide contact information for a data access committee, ethics committee, or other institutional body to which data requests may be sent. If data are owned by a third party, please indicate how others may request data access.

7) Please amend your detailed Financial Disclosure statement. This is published with the article. It must therefore be completed in full sentences and contain the exact wording you wish to be published. 1) State what role the funders took in the study. If the funders had no role in your study, please state: "The funders had no role in study design, data collection and analysis, decision to publish, or preparation of the manuscript." 2) If any authors received a salary from any of your funders, please state which authors and which funders.. If you did not receive any funding for this study, please simply state: u201cThe authors received no specific funding for this work.u201d  
**Reviewers' comments:  Reviewer's Responses to Questions**

Comments to the Authors:

**Reviewer #1:** The authors present a thorough analysis for how different isoforms of tropomyosin in budding yeast mechanistically maintain and organize actin cables. They utilize novel tagged, yet functional proteins for both isoforms of tropomyosin for this analysis. They found that the different isoforms are able to bind actin cables indepenedent of whic formin nucleated the cable, this finding is distinct from previous reports done in other organisms, showing that there is diversity in actin-binding protein function across species. They reveal novel functions for Tpm2, in that it, but not Tpm1 can regulate retrograde actin cable flow, and that Tpm2 can function as the sinlge copy of tropomyosin, as long as it is expressed in sufficient quantities. They also show that the concentration of tropomyosin in the cell, and not a specific isoform, is responsible to maintaining the balance between actin networks. **The manuscript presents strong evidence for their claims and their conclusions match the data presented. This work is a significant step forward for the cytoskeletal field and presents novel tools that can be an important contribution to other labs in this area of research.**

We sincerely thank the reviewer for their positive assessment of our work and the insightful suggestions. We have now incorporated all the suggestions which has greatly benefitted the final manuscript.

**Minor Issues:**

1) Define actin binding protein as (ABP) in the abstract, as the authors use the abbreviation in the introduction without making the distinction.

We thank the reviewer for pointing this out. We have now defined “actin-binding protein” (ABP) in the abstract from the outset for clarity.

  2) Move the OM45-mCherry images from Figure S1I to Figure 1 to match with Figure 1D. Having the images and subsequent analysis on different figures is very awkward.

We thank the reviewer for this suggestion and agree that presenting the images and their corresponding quantification together is much clearer. We have now moved the images for mitochondrial morphology to **Fig. 1D,** and its quantification is represented in **Fig. 1E** in the revised manuscript.

  3) Please report n values for charts in Figure 1C and 1D.

We apologize for this omission. We have now added the “n” values for these charts in the **Fig. 1 Legend** in the revised manuscript.

4) In Figure 2 and S2, the authors utilize phalloidin staining as a readout with deletion of formins. They state that their tagged tropomyosins were unstable when fixed, thus separate analysis. However, they have ymScarleti-AS-Tpm1 later in the figure and LifeAct-eGFP later in the manuscript, so co-expressing these together would allow for simultaneous analysis without fixation.

We thank the reviewer for this suggestion. While this is possible, but we decided against this because LifeAct is known to have expression-dependent artefacts *in vivo* (Courtemanche et al 2016) and compete with other actin-cable binding proteins, which could significantly affect actin cable dynamics and potentially mask/convolute subtle changes in cable properties in mutant strains. In addition, while ymScarleti-^AS^Tpm1 could be detected in live cells, its signal was weaker and much more prone to photobleaching, limiting its experimental utility in comparison. For the above listed reasons, we did not use co-expression of LifeAct for our analysis.

 5) There is no quantification for Figure 2G with the colocalization of the 2 isoforms, so while they have a single image, quantification of the colocalization would strengthen the argument by showing that colocalization is a common occurance.

We thank the reviewer for this suggestion. To strengthen our claim, we have now performed a quantitative colocalization analysis for ymScarleti-^AS^Tpm1 and mNG-^AS^Tpm2. The results, presented in the new **Fig. 2H**, show a high Pearson’s correlation coefficient, providing robust quantitative support for our qualitative observation that the two Tpm isoforms indeed colocalize on actin cables.

6) In Figure 4 with the TIRF analysis, the authors utilize yeast cofilin and the yeast tropomyosins, but then use human profilin. Budding yeast profilin is easily purified and functions with actin in TIRF, and while human profilin can partially compensate for a budding yeast profilin mutant, it would be a cleaner experiment using budding yeast profilin, just to eliminate any variables.

Thank you for this insightful comment. We agree that human profilin can only partially compensate for yeast profilin *in vivo*. However, it's important to note that in the *in vitro* experiments presented here, human profilin was only present during the initial filament elongation phase, prior to the filaments' exposure to yeast cofilin and/or Tpm. Therefore, the specific source of profilin (human or yeast) during filament elongation should not influence the results presented in **Fig. 4**, which focus on the effects of cofilin and Tpm on actin filaments (when no profilin is present in the system).

 7) In the text about Tpm2 mRNA levels on page 8, the authors cite their Figure S4F, when it relates to the data in Figure S4E.

We are sorry for this error and thank the reviewer for pointing this out. We have now correctly cited **Fig. S7C**, when referring to Tpm2 mRNA levels in revised manuscript.

  8) In the Discussion section on page 13, the authors talk about the LatB experiment, but then cite Figure 4G and 4H, when the data is in Figure 5D and 5E.

We apologise for this error and thank the reviewer for pointing this out. We have now correctly cited **Fig. 5D** and **5E**, when referring to the LatB sensitivity experiment in the revised manuscript.

 9) In the Discussion section on page 14, the authors talk about the LifeAct-eGFP actin patch experiments, but then cite Figure 5E-G, when the data is in Figure 6E-G.

We apologise for this error and thank the reviewer for pointing this out. We have now correctly cited **Fig. 6E-G**, when referring to the actin patch experiments in the revised manuscript.

We sincerely apologize for these citation errors and thankful to the reviewer for catching them. We have meticulously corrected all figure citations throughout the manuscript to ensure accuracy.

**Reviewer #2:** The manuscript by Dhar *et al.* investigates the differences between the two budding yeast tropomyosin homologs, Tpm1 and Tpm2. The authors utilise a recently published fluorescently tagged version of Tpm1 and Tpm2 to explore their localization and functionality. This work is relevant to the field as it contributes to our understanding of the specific roles of tropomyosin homologs in budding yeast cells and may shed some light on the multiple isoforms found in higher eukaryotes. The *in vitro* binding assays, which demonstrate comparable protection of actin filaments against cofilin severing by purified Tpm1 and Tpm2, suggest a degree of functional redundancy between the two homologs.

While the research presented holds potential merit, I found certain aspects of the manuscript challenging to understand, which hindered a full appreciation of the work. To enhance clarity and ensure the work is accessible to a broader readership, I recommend addressing the following points:

We sincerely thank the reviewer for their thoughtful feedback and detailed suggestions. We have performed a comprehensive revision, incorporating the proposed changes which has enhanced the manuscript's clarity, logical presentation, and overall readability. We believe these revisions have significantly strengthened the study and are thankful for the reviewer's input.

**Experimental Design and Justification:** It would be beneficial to include a more explicit rationale for the design of specific experiments. Clearly articulating the reasoning behind each experimental approach would strengthen the overall logical flow of the manuscript.

We thank the reviewer for this suggestion. We have now added more detail and rationale for our experimental design and approach to improve overall clarity in the manuscript.

**Genetic Background Details:** Related to the above point, providing a more thorough justification for the specific genetic backgrounds used in the study would help readers understand their relevance to the experimental questions.

As per the reviewer’s suggestion, we have now added details of genetic backgrounds used in our study for each experiment in the text wherever applicable.

**Methodological Context:** Given the reliance on a recently published method for tagging tropomyosins, a brief background or more detailed description of this method would be helpful for readers who may not be familiar with it in the introduction. This would allow for a more comprehensive understanding of the experimental setup.

We agree and thank the reviewer for this suggestion. We have added relevant background on the development of mNG-Tpm fusion proteins, detailing the inclusion of a 40-amino acid flexible linker which is key to their functionality, for more clarity in the Introduction Section **(Lines 45-52)**.

**Clarity and Precision in Terminology:** There were several instances of typos and references to the wrong figures. Fixing these mistakes would significantly enhance readability and prevent potential misinterpretations. A careful review of all figures and text for accuracy is necessary.

In summary, a focused revision on the clarity and logical presentation of the writing would greatly improve the manuscript's accessibility, allowing both expert and less expert readers to fully understand the significance of the work and critically evaluate the results.

Below, I provide examples of specific sections that could benefit from revision. Please note that this list is illustrative, and a comprehensive review of the entire manuscript for clarity and justification is recommended.

We apologise for the errors in the original manuscript and thank the reviewer for pointing this out. We have now corrected all errors regarding figure citations in the text and have added more background and explanation as suggested by the reviewer in the revised manuscript to improve clarity and overall flow. The details of many of the changes are also mentioned in our responses other than individual points raised by the reviewers above and below.

**Major concerns:**

1- The title does not represent the findings. It is inaccurate to write “formin-independent” as Tpm1 and Tpm2 bind actin cables nucleated by formins Bnr1 and Bni1. You mean to say that the “localization of Tpm1 and Tpm2 is agnostic of formin type”. Please revise the title for clarity.

We agree and thank the reviewer for this suggestion. We have now modified the title as “**Functional redundancy and formin-isoform independent localisation of Tropomyosin paralogs in *Saccharomyces cerevisiae***”. The new title correctly communicates our findings that the localisation of Tpm1 and Tpm2 on actin cables is agnostic of the which formin isoform nucleated the cable.

2- The introduction lacks relevant details about previous experiments that tested the localization and functionality of Tromomyosins. This background information is critical to understand how your approach is different and why it may successfully demonstrate homolog specific functions of tropomyosins. For example, what’s the main difference that makes these fluorescently tagged tropomyosins functional? How was functionality tested before? Growth assays, quantitative imaging of the actin cytoskeleton, functional assays of the actin cables…?

We thank the reviewer and have added additional background literature in the Introduction **(Lines 45-52)** and Discussion regarding Tropomyosin tagging and the functionality of tagged constructs **(Lines 425-439)**. While many studies have used tagged Tropomyosin to track actin filament populations in cells, the tagged protein was often expressed as an extra copy alongside the native Tpm, or the isoform studied was non-essential - leaving functionality untested. The question of functionality was addressed by Brooker et al. (2016), which tested *S. pombe* Tpm Cdc8 and found that C-terminal tagging rendered it non-functional, while N-terminal tagging was only partially functional: it could partially rescue growth and support contractile ring formation, but in an abnormal manner. This suggests that although the N-terminus is more amenable to tagging, direct attachment still disrupts normal regulation, likely by hindering head-to-tail Tpm dimer contacts and interfering with binding of actin-associated proteins such as myosin-V. In contrast, our approach uses a 40-amino-acid flexible linker to separate the N-terminal fluorophore (mNeonGreen) from Tpm, potentially preserving head-to-tail dimer contacts and allowing for the good *in vivo* functionality we observe.

3- The authors emphasize the improved capability of the ”AS” versions of Tpm1 and Tpm2 to rescue the length and number of actin cables of *∆tmp1* mutant cells over the non-AS versions with data from Figure 1 supporting this difference. However, the data in figures S1C, D-F and I show no differences between the AS and non-AS versions in rescuing growth, actin cables and mitochondria distribution. The discussion doesn’t address this discrepancy. A simple explanation acknowledging that a full complement of full-length actin cables appears to not be necessary for function.

We would like to clarify to the reviewer that while we observed a similar rescue in growth of *∆tpm1* cells expressing mNG-^AS^Tpm1 or mNG-Tpm1 **(Fig. S1C, S1E, S1F, S2C, S2D)**, the major difference between mNG-Tpm1 and mNG-^AS^Tpm1 was observed in the actin cable rescue in ∆*tpm1* cells **(Fig. 1B, 1C)**. Expression of mNG-^AS^Tpm1 restored full length actin cables, while mNG-Tpm1 only restored partial length actin cables in the mother cell **(Fig. 1B, 1C, S1G)**, suggesting an improvement in actin protective capabilities by the addition of -AS- dipeptide. Interestingly, while growth **(Fig. S1C, S1D)** and mitochondrial morphology **(Fig. 1D, 1E)** seem to be near completely restored to wildtype levels with both constructs, it suggests that these cable-dependent functions do not require the presence of full-length actin cables. The sufficient condition to majorly drive these actin-cable dependent processes may be the presence of normal and polarised actin cable architecture towards the bud, while actin cable length variations are well tolerated by cells. These results are described in the Results and Discussion section, and we have now added more discussion on this aspect in the revised manuscript **(Lines 441-453).**

4- The authors hypothesize that Tpm1 and Tpm2 do not display a bias for actin filaments nucleated by Bnr1 or Bni1. Although I don’t believe that Tpm1 and Tpm2 only bind to either Bni1 or Bnr1 actin cable networks based on the protein distributions shown in Figure 1A and B, the data used to support this lack of bias is not convincing.

First, to test this hypothesis, the authors visualize the localization of mNG-AS-Tpm1 and mNG-AS-Tpm2 in *∆bnr1* and in *∆bni1* cells. The rationale for using this approach to test their hypothesis is missing from the results. It is also impossible to discern the actin decorated cables above the green background in figures 2A and D. Using a black and white inverted lookup table may help. Finally, the authors overlook the possibility that removing one of the two main actin cable networks with the deletion of the formin responsible for their nucleation may alter the distribution of the Tpms. This possible interpretation is missing from the discussion.

We acknowledge the reviewer’s concern and would like to clarify that we have reached the conclusion that Tpm1 and Tpm2 indiscriminately localise to actin cables nucleated by either formin isoform, Bnr1 and Bni1, through multiple lines of evidence and thus, we are confident that our data largely supports it.

Firstly, we assessed mNG-^AS^Tpm1 and mNG-^AS^Tpm2 localisation in wildtype, *∆bnr1* and *∆bni1* cells. Our hypothesis was that if Tpm1 and Tpm2 preferably localised to actin cables nucleated by any one of the formin isoforms, their localization would be significantly affected when that formin isoform is not present in cells as opposed to the other isoform. But, our measurements of Tpm-bound actin cable number and Tpm-bound actin cable length revealed no significant loss of localisation of either Tpm1 or Tpm2 in *∆bnr1* and *∆bni1* cells as compared to wildtype **(Fig. 2A-F, S3A-F, S4)**, suggesting that Tpm1 and Tpm2 do not show preference for actin cables made by a particular formin isoform.

Now, the reviewer has raised an important question whether the preference may be shown only when both formins are present in cells, and that the localization preference may itself change when either of the formin isoform is absent. This is a possibility and unfortunately not possible to test through the above experiment. However, our data from other experiments suggests against this possibility: **(i)** The localisation of mNG-^AS^Tpm1 and mNG-^AS^Tpm2 is very identical in both the mother and daughter compartments in wildtype cells (**Fig. 1A, S3G, S3H); ii)** Dual colour imaging of ymScarleti-^AS^Tpm1 and mNG-^AS^Tpm2 also shows that these two isoforms co-localize very strongly on actin cable structures in both the bud and mother compartment in wildtype cells **(Fig. 2G, 2H);** **(iii)** The ratio of mean bud/mother fluorescence signal for both mNG-^AS^Tpm1 and mNG-^AS^Tpm2 is similar in wildtype cells, and changes in the same manner in *∆bnr1* and *∆bni1* cells **(Fig. S3G)**. The above three observations strongly suggest against the possibility that Tpm1 and Tpm2 may show a preference for a particular formin isoform only in wildtype cells when both formin-isoform made actin cables are present. We have now discussed this possibility in the Results section in the revised manuscript **(Lines 172-188)**.

Secondly, we also assessed the localization of mNG-^AS^Tpm1 and mNG-^AS^Tpm2 in mating haploid wildtype/∆*bnr1* cells and found that both mNG-^AS^Tpm1 and mNG-^AS^Tpm2 localise to the mating projection with similar spatiotemporal kinetics in both wildtype and ∆*bnr1* cells (**Fig. 3A, 3C)**. Since, it is well known that Bni1 is the major formin responsible for nucleation of actin filaments at the mating projection, this observation also suggests that Tpm1 and Tpm2 indiscriminately bind to actin filaments made by either formin Bni1.

            Second, co-expression of ymScarleti-AS-Tpm1 and mNG-AS-Tpm2 was used to show colocalization of both tropomyosins to the same actin cable network. However, figure 2G shows little colocalization of the two tropomyosins. Indeed, there is more distinct localization (green and purple signals) than co-localization (white signal) between the two proteins.

As per the reviewer’s suggestion we have now quantified Pearson’s correlation coefficient to quantitatively depict co-localization of ymScarleti-^AS^Tpm1 and mNG-^AS^Tpm2 **(Fig. 2H)**. This analysis also suggests a high degree of colocalization between the two proteins.

Finally, the data shown in figures 1A and B show the localization of each Tpm1 and Tpm2 constructs to both Bni1 and Bnr1 networks and could be used to support the lack of bias of each tropomyosin for a specific actin cable network. Some enlarged panels with arrows labeling the important actin networks may help illustrate the lack of bias.

Thank you for this suggestion. We have now added new magnified images as additional panel in **Fig. S3G** to emphasise the localization of Tpm1 and Tpm2 to different actin cable networks in both bud and mother cell compartments in wildtype cells.

5- The authors propose that the low endogenous expression level of Tpm2 is responsible for its inability to rescue *∆tpm1* phenotypes. This is based on the in vitro experiments that show that purified Tpm1 and Tpm2 can both protect actin filaments from cofilin mediated severing. They show in Figure S4 that overexpression of Tpm2 can indeed rescue the presence of actin cables and the viability of *∆tpm1* cells. The authors claim that actin cable number and length are rescued but no data supports these quantitative measurements.

We thank the reviewer for pointing this out. We had concluded this result qualitatively from the data shown in **Fig. S5B** along with a quantification of the percentage of cells which showed rescue in actin cables **(Fig. S5C),** but we have now quantified and added quantitative analysis of actin cable number and length to robustly support our conclusions. The new data is presented in **Fig. S5D, S5E**.

             In figure S4E, how can the fold change of Tpm2 transcript be the same as wildtype when the endogenous transcript is present in addition to the exogenous ^pTpm2^TPM2 expressed from a plasmid?

We agree with the reviewer’s observation, and we had hypothesised that this is due to the weak strength of the Tpm2 promoter. Hence to test whether this discrepancy is real, we have now repeated the RT-qPCR experiment. In our new results, we are now able to detect a ~2.5 and ~3 fold significant increase in Tpm2 mRNA levels in the cells containing both ^pTpm2^Tpm2 and ^pTpm1^Tpm2 low-copy number plasmid, respectively, as compared to the wildtype and control strain carrying empty plasmid. We have now replaced the previous results in **Fig. S7C** with the new data.

            Finally, the authors mention that even a mild overexpression is sufficient to rescue the phenotypes. Can you clarify what is the minimal amount of Tpm2 you found is sufficient to rescue the *∆tpm1* mutant phenotypes?

Based on our new RT-qPCR analysis shown in revised **Fig. S7C**, the minimal amount of Tpm2 that we found to be sufficient to rescue *∆tpm1* is ~2.5 times the endogenous Tpm2 levels at the mRNA level. Assuming an equal conversion of this mRNA through translation, ~2.5 times more Tpm2 protein is required to rescue mutant phenotypes in *∆tpm1* cells, suggesting our conclusion that Tpm2 is maintained at limiting levels in cells.

6- Clarify the level of expression for the data shown in Figure 6. Which of these data were acquired in cells with high copy plasmid and which ones from cells with low copy plasmids?

We apologise for the confusion. All the plasmids used in **Fig. 6** are low-copy number centromeric plasmids.

As per Reviewer 3’s suggestion, we have now adopted a new plasmid nomenclature to include the copy number information of the plasmids, which is also described in the Materials and Methods Section. Please see our response below to Reviewer 3 (last query).

7- I’m curious to know why the mNG-AS-Tpm1 and mNG-AS-Tpm2 constructs were not integrated into the endogenous *tpm1* and *tpm2* loci if they are fully functional. Why were they expressed from plasmids or integrated into the leu2 loci?

We had described these fusion proteins originally in *Hatano et al. 2022 (JCS)* and used the native promoters of *tpm1*/*tpm2* and the routinely used *leu2*-based integration vectors (*pRS305*) for expression. Thus, we characterised the same strains and continued to use the same vectors for this study. Also, since mNG-^AS^Tpm2 was found to be functional only at higher expression levels **(Fig. S2C, S2D)**, it was necessary to use it as an extra copy in addition to the endogenous Tpm2 gene. However, we completely agree with the reviewer that endogenous tagging will beneficial and we will aim to create endogenously expressing mNG-Tpm1 strains for future studies.

**Other comments on the figures:**

1. Figure 1A and B (and S1A and B and 3A and B). Are these three rows of images a timelapse of the same cell? The specific timestamps on each time point are missing.

Yes, these are time-lapse images of the same cell. Thanks for pointing this out. We have now added the time stamps in the respective panels.

2. Figures S1A and B. The images are too small to discern the fluorescent signal.

Thanks for pointing this out. We have now split the original **Fig. S1** into two supplementary figures (**new Fig. S1 and S2**) for better reading and clarity in the revised manuscript.

3. Figure S1C. What’s the shaded area around each curve? SEM? This data also lacks statistical test to determine whether there is any significant difference between the curves especially *∆tpm1 :: mNG-Tpm1* and *∆tpm1 :: mNG-AS-Tpm1.*

The shaded area around the curves is the Standard Deviation while the solid line represents the mean. We have now mentioned this in the figure legend. We have now also calculated generation time from the growth curves and performed a statistical analysis to determine if the two strains have significant difference in growth rate. Our analysis suggests that there is no significant difference in generation time between *∆tpm1 :: mNG-Tpm1* and *∆tpm1 :: mNG-^AS^Tpm1.* This new data is presented as a table in **Fig. S1F** in the revised manuscript.

4. Figure S1. The legends for panels H and I are missing.

We are sorry about this omission. We have now added legends for these panels and corrected all errors regarding figure citations in the revised manuscript.

5. Figures 2A and D. It is not possible to discern the actin decorated cables above the green background in these images. Using a black and white inverted lookup table may help.

Thanks for this suggestion. We have now changed the LUT in these images and the new images are presented in revised **Figure 2A, 2D**. We have also used arrows to denote Tpm-bound actin cables for improved readability.

6. Figure S2G. The lines are too thick and the markers can’t be distinguished from each other.

We have modified and replaced the graph for better visual clarity, now presented as **Fig. S3H** in the revised manuscript.

7. Figure S5C and E. Can you provide an explanation for the discrepancy between the trends seen in C versus the trend seen in E.

This discrepancy likely arises due to a greater number of cells being included in the intensity quantification which have a lower expression of mNG-^AS^Tpm2 since the copy number of the high-copy pRS425 plasmid varies a lot from cell to cell. The cells with higher expression usually tend have a saturated fluorescence intensity and hence, would get excluded from the intensity quantification, bringing the mean of the population lower when compared to a western blot analysis.

**Minor comments (this list is not comprehensive):**

1- Line 18. ABP acronym has not been defined. It is defined twice at lines 378 and 440.

2- Line 228. Shouldn’t it say ^pTpm1^TPM2 instead of ^pTpm1^TPM1?

3- Line 279. “We used exogenously expressed…” not “exogenous”.

4- Lines 430 and 431. Refers to figures 4G and H. There are no Figures 4G and H.

5- Line 446. Refers to Figures 5E-G. There are no Figures 5F and G.

We have corrected these and other minor errors that we found after a comprehensive reading of the manuscript. We thank the reviewer for their suggestions on our manuscript, which have been a significant addition to the study.

**Reviewer #3:** This manuscript from Dhar and colleagues addresses the role of two yeast tropomyosin isoforms, Tpm1 and Tpm2, in the regulation of actin cable architecture and function in budding yeast S. cerevisiae. They created novel mNeonGreen-tagged acetylation-mimic (AM) Tropomyosin probes expressed as the sole gene copy at the endogenous loci for both Tpm1 and Tpm2 that complemented cell growth and actin cable architecture in vivo. These new fluorescent probes for visualizing tropomyosin decoration of actin cables allowed the group to show Tpm1 and Tpm2 decorate cables generated by either formin Bni1 or Bnr1. Further, Tpm1 and Tpm2 bind actin cables indiscriminately in cells undergoing mating-induced fusion or miotic growth and division. The group used purified proteins to demonstrate that Tpm1 and Tpm2 equally protected actin filaments from cofilin-mediated severing. They also show that contrary to previous reports, increased expression of Tpm2 complements the cell growth defects and actin cable architecture of tpm1D, demonstrating that Tpm1 and Tpm2 are more functionally interchangeable than previously thought. Interestingly, even mild (2-fold) overexpression of Tpm2 is sufficient to suppress the loss of actin cables in tpm1D. Moreover, they show that actin cables in tpm2D cells are hyper-sensitive to latrunculin, which represents one of the first phenotypes found for tpm2?. Lastly, the group found that Tpm2 overexpression not only restores actin cables in tpm1D cells, but also rescues defects in mitochondrial morphology, secretory vesicle polarization, and endocytic actin patch dynamics. These results conclude in a model showing that Tpm1 and Tpm2 co-occupy actin cables generated by either yeast formin Bni1 or Bnr1 and that elevated Tpm2 levels suppress tpm1D actin-related phenotypes.

**Validity**

**This manuscript utilizes multi-prong approach combining powerful yeast genetics, quantitative live and fixed-cell imaging as well as in vitro reconstitution using purified proteins. This multi-faceted approach to understanding the mechanistic basis for yeast tropomyosin function in vivo is well-rounded.** The genetic data in this paper are robust, and conducted in a rigorous manner. The authors use modern quantitative measurements of actin cables (McInally et al., 2022) in their analyses of both phalloidin-stained and Tpm-decorated actin cables.

**Significance**

1. **This manuscript contains a number of significant findings that will be well received by the cell biology and biochemistry fields.** The generation of functional endogenously-tagged tropomyosins in vivo provides a powerful tool for the yeast actin field to dissect Tpm1 localization and function beyond this study. Before this manuscript, a previous paper (Drees et al., 1995) reported that although deletion of TPM1 and TPM2 together was lethal in yeast, overexpression of Tpm2 could not rescue tpm1D. Instead, Dhar and colleagues show that this result was not correct, and that Tpm2 overexpression restores actin cables and other functions in tpm1D. Uncovering how yeast utilize two tropomyosin isoforms will appeal to a broad audience in the actin field, since higher order eukaryotes typically possess multiple tropomyosins.

We thank the reviewer for their positive assessment of the impact of our study. We are also grateful for the reviewer’s in depth reading and critical feedback which has helped us to significantly strengthen our study after revision.

**Data and methodology**  The quality and presentation of the cell biological, biochemical and genetic data is sound overall. However, the actin cable quantification methodologies require some additional explanation for readers. For quantification of actin cable length and number, the authors simply cite McInally et al., 2022 and show an example image analysis pipeline. However, McInally et al., 2022 only demonstrates a quantitative analysis pipeline for actin cable length, not cable number. Additionally, the authors claim that fixation disrupts tropomyosin decoration with actin cables and thus forced them to measure Tpm1/Tpm2 coated cables from live cell images as a population average. No methods were provided to explain how they determined Tpm1-coated cable length and number, as this was not provided in McInally et al., 2022.

We thank the reviewer for bringing our attention to this point. We would like to clarify that the actin cable length analysis automatically gives us the actin cable number as well and is part of the workflow in *McInally et al., 2022* as we simply count all the cables that we annotate for measuring length. We have now mentioned this in the Material and Methods Section of the revised manuscript for clarity.

In our experience, fixation of cells expressing mNG with 4% PFA caused a substantial reduction in Tpm signal, and the images were of lower quality as compared to that of live-imaging of mNG-Tpm. This could be due to effect of the fixative on Tpm-binding to cables or the change in behaviour of the mNG fluorescent protein post-fixation. Thus, to overcome that problem, we chose to measure cable parameters for mNG-Tpm from images of live-cells instead of fixed cells. The Tpm-bound actin cable length and number were measured in the same manner as the actin cable length and number as described by *McInally et al. 2022*. We have mentioned this information in the Materials and Methods Section of the manuscript **(Lines 607-613)**.

3. **Analytical approach**

The authors provide extensive quantitative analyses wherever appropriate in each figure. However, I do have a concern about graphs in Figure 2B and 2E, in measuring actin cable length differences in various formin mutant backgrounds. By eye, there does not seem to be appreciable differences between the data means and distributions, yet the authors claim bnr1D cells have significantly longer cables. In addition to the graphs not appearing correct by eye, bnr1D cells would be expected to have shorter cables, since they lack the formin responsible for generating long, thick cables in the mother cell compartment. Further explanation or speculation is needed on this.

We are grateful to the reviewer for raising this important point. While Bnr1 is indeed critical for nucleating robust cables in the mother cell, our observations suggest that overall cable length is not the primary parameter affected by its deletion. This is consistent with recent studies demonstrating that actin cables scale with cell size (*McInally et al., 2020, 2023)*. In the Δ*bnr1* background, the remaining, thinner cables still elongate to match the cell's dimensions due to the scaling law. The loss of Bnr1 or Bni1 primarily causes a decrease in actin cable number which is consistently seen in our data and is in line with the established role of these formins in actin cable nucleation (*Buttery et al., 2007)*.

The reviewer's comment led us to revisit our statistical analysis. Recognizing that this data may not follow a normal distribution, we switched from a One-Way ANOVA to a Kruskal-Wallis test followed by Dunn's multiple comparisons test. This more suitable analysis confirms that the cable lengths in Δ*bnr1* cells are not significantly different from wildtype. However, we have observed that Δ*bnr1* cells tend to have somewhat elongated morphology which might lead to longer cable lengths due to the phenomenon of actin cable scaling with cell size. We have now updated **Figures 2, S3, and S4** accordingly with the updated statistical analysis.

4. **Suggested Improvements**  · In Figure 1A, the authors claim that the acetylation mimic tropomyosin constructs yield clearer and brighter cable staining than the non acetylation mimic constructs shown in Figure S1A. To make this claim, it would be more convincing to show each construct side-by-side with equally contrasted images acquired with identical settings. A fluorescence quantification of cables would also help this be more convincing.

As per the reviewer’s suggestion, we have now added images of cells expressing -AS and non-AS containing mNG-Tpm1/Tpm2 proteins acquired with identical settings along with a quantitative analysis of signal-to-noise ratio for mNG cable signal. The data is presented in the revised manuscript as **Fig. S2A, S2B**. This analysis reveals a statistically non-significant increase in signal-to-noise ratio for mNG-^AS^Tpm1 as compared to mNG-Tpm1, while a statistically significant increase in signal for mNG-^AS^Tpm2 as compared to mNG-Tpm2, suggesting an improvement in SNR after -AS- addition for mNG-Tpm2.

  · In Figure 1D, the authors quantify mitochondrial fragmentation as a product of actin cable function. However, the authors fail to properly introduce why this is a viable assay for quantifying cable function. A brief explanation and citing a previous work would be beneficial to the reader. Also, the authors did not call out Figure S1I as the representative mitochondrial images to accompany the graph in Figure 1D.

We thank the reviewer for pointing out this ambiguity. Mitochondrial morphology and inheritance are known to be dependent on actin cables in budding yeast; however, the exact molecular mechanisms of this control are still unknown. Previous work has shown that actin cables can co-localize with mitochondria and interact directly with mitochondria via certain adaptor proteins and may contribute to stability of mitochondrial tubules (*Boldogh et al. 1998 (JCB), Fehrenbacher et al. 2004 (Cur. Bio.), Chernyakov et al. 2013 (Cur. Bio.)*). It has been shown that the loss of Tpm1 leads to fragmented mitochondrial morphology, and this may be due to the loss of actin cables caused by absence of Tpm1. Thus, testing mitochondrial morphology is one of the viable assays that can be used for testing mNG-Tpm functionality and cable function. We have also corrected the errors in figure citations related to this data and have added the above-mentioned relevant background in the Results section in the revised manuscript.

  · For quantifying Tpm-bound cables, the authors revert to using mNG-AS-Tpm constructs expressed as a second copy from a separate locus in the genome while endogenous unlabelled Tpm1 is simultaneously being expressed. This seems odd, since the take-home message from Figure 1 was the functionality of these new endogenously expressed mNG-AS-Tpm constructs. Could the authors provide some rational for this deviation in the text and potentially in the methods section.

We would like to clarify to the reviewer that we measured Tpm-bound actin cable length and cable number in both cells expressing mNG-^AS^Tpm1 as an additional integrated copy in the genome at the *leu2* locus **(Fig. 2A-C)** and as the sole copy where endogenous Tpm1 is not present **(Fig. S4)**. Both experiments supported the conclusion that Tpm1 localization to actin cables is not biased for cables made by either formin Bnr1 or Bni1. For mNG-^AS^Tpm2, we performed this analysis only expressing it as an extra copy **(Fig. 2D-F)** since mNG-^AS^Tpm2 did not show functionality when expressed from its native promoter at levels comparable to its endogenous expression **(Fig. S2C, S2D)**. Additionally, we also observed that when expressed as a sole copy, mNG-^AS^Tpm1 did not decorate actin cables along their entire length as compared to when expressed in the presence of the endogenous untagged Tpm1 protein, probably due to heterodimer formation with the endogenous Tpm1, which we have now discussed in the revised manuscript in **Lines 197-202, 462-471**.

· In Figure 2C there is no difference in Tpm-bound cable number between bnr1D and bni1D cells expressing mNG-AS-Tpm1, but in Figure 2F there is a difference in between Tpm-bound cable number between bnr1D and bni1D cells expressing mNG-AS-Tpm2. The authors do not provide any explanation in the text for this discrepancy.

We thank the reviewer for drawing our attention to this. We carefully evaluated the data, and the discrepancy seems to be due one of the biological replicates in **Fig. 2F** having higher cable numbers for ∆*bni1* cells as compared to the other two biological replicates, which could be due to natural heterogeneity or experimental stochasticity. Thus, we quantified more cells in this experiment and have presented the updated data in **Fig. 2F** in the revised manuscript. With the addition of new data points, there is still a significant but minor difference between cable numbers among ∆*bni1* and ∆*bnr1,* most likely a result of natural heterogeneity within our experiments. However, we do consistently find that cable numbers significantly differ from wildtype when Bnr1 or Bni1 is not present, as expected due to the established role of these proteins in actin cable nucleation and elongation.

 · In Figure 3, the authors do not describe how they mark the onset of fusion. A more detailed methods section for mating and fusing yeast would be helpful.

As per the reviewer’s suggestion, we have now updated the methods section for the mating experiment in the revised manuscript. The fusion time point was determined to be the one where fluorescence intensity appeared in the diffused over to the other mating yeast cell which originally did not have any fluorescence. This is evident in the time-lapse images depicted in **Fig. 3A, 3B.** The fusion time point was set as t=0 for the quantitative analysis shown in **Fig. 3C, 3D**.

  · In Figure 3A and Figure 3B, it is very difficult to discern visible Tpm-coated cables in the mating yeast. Clearly, the expression levels decrease as quantified in Figure 3C and Figure 3D, but it remains unclear to me how well Tpm decorates cables in the mating yeast based on the provided representative images.

Thank you for bringing this to our attention. We have now represented deconvolved images and used inverted grays LUT for better visibility of Tpm-coated actin filaments during yeast cell-cell fusion. We did not use deconvolved images previously because we were more interested in mNG-Tpm localization at the strong actin dense foci formed at the mating tip which was apparent without the need for deconvolution. The actin foci at the mating projection has been reported in previous studies in *S. pombe* and is driven by formin-mediated actin assembly (*Dudin et al., 2015 (JCB)*). Other than the strong localization at the fusion foci, we do observe Tpm decoration on longer actin cables in mating yeast as well. The new images are presented on **Fig. 3A, 3B** in the revised manuscript.

  · There are inconsistencies in data sets between Figure S2 and Figure S3 that are not described in the text:  o In Figure S2, cable length analyses in wildtype, bnr1D and bni1D cells differ depending on whether mNG AS-Tpm1 or mNGAS-Tpm2 are expressed.

We appreciate the reviewer’s point. We believe that this discrepancy arises mainly due to natural heterogeneity in the experiments and variations in staining efficiency among replicates. However, even though differences may be statistically significant, cable lengths do not vary much from wildtype in ∆*bnr1* and ∆*bni1* cells as the other formin is still present to nucleate and elongate cables. In fact, recent studies have shown that actin cable length scales with cell size in *S. cerevisiae (McInally et al., 2020, 2023).* What is more robustly observed throughout our data **(Fig. 2, S3, S4)** is the significant drop in actin cable number when either Bnr1 or Bni1 is absent from cells.

In Figure S3, bni1D cells contain significantly longer actin cables compared to bnr1D cells specifically when using phalloidin staining instead of measuring mNG AS-Tpm1 signal along cables.

We thank the reviewer for bringing this to our attention. This discrepancy may be caused due to the following reason:

In the data shown in **Fig. S3**, mNG-Tpm1 fusion proteins is expressed the sole Tpm1 copy in cells and endogenous Tpm1 gene is deleted. It is possible that these fusion proteins show better binding in the presence of the endogenous untagged Tpm proteins, due to the possibility of heterodimer formation between tagged and untagged protein monomers, which would have less hindrance in binding to actin cables as compared to a homodimer of tagged Tpm monomers. These reasons could affect mNG-Tpm decoration on actin cables and hence, also contribute to lower cable lengths when measured for mNG-Tpm signal. We have now discussed this observation in the revised manuscript **(Lines 197-202, 462-471)**.

o These inconsistencies make it difficult to interpret the role of each formin in regulating actin cable architecture.

We thank the reviewer for bringing our attention to these discrepancies and we have now discussed these observations and their possible underlying reasons in the revised manuscript **(Lines 158-172)**, which improves our understanding of the use of mNG-Tpm proteins in cells and the role of formin proteins in actin cable nucleation.

  · For Figure 4, the authors do not mention that their results (about Tpm1 and Tpm2 protecting actin filaments from cofilin-mediated severing) potentially conflict with a previous study from Fan et al., 2008. This will help clarify things for the field.

We apologize for omitting the citation to *Fan et al., 2008*. We appreciate you bringing this to our attention. Our findings corroborate their work, as we also observed that yeast Tpm1 does not provide complete protection against yeast cofilin-1 mediated severing. Furthermore, our study extends these findings by comparing the protective capabilities of Tpm1 and Tpm2, which showed similar protection from cofilin-1, and by including human Tpm1.7, which offered superior protection. We have now incorporated a brief discussion comparing our results with those from *Fan et al., 2008* in the revised manuscript **(Lines 507-514)**.

 · In the text describing Figure S4A-C, the authors detail how their Tpm2 overexpression experiments differ from Drees et al., 1995. The authors claim that the previous work, which expressed Tpm2 on a high-copy plasmid under control of a galactose-inducible promoter, likely generated a lethal dosage of Tpm2, leading to no rescue of tpm1D actin phenotypes. While this is very plausible, given that a mild overexpression of Tpm2 shown in this paper can suppress tpm1D actin phenotypes, it would be significantly more convincing to do a side-by-side comparison using the same plasmid-promoter system Drees et al., 1995 used along with the plasmids used in the current study. I realize that this is asking for more experiments where the answer from the data in the present study is already clear. However, doing them all side-by-side will lay the issue to rest once and for all, and provide final clarity on the genetic relationship between Tpm1 and Tpm2.

As per the reviewer’s suggestion, we have now tested the possibility that whether the strong galactose-based over expression of Tpm2 could have caused *Drees et al. 1995* to conclude that Tpm2 over expression could not rescue phenotypes in *∆tpm1* cells. Similarly to *Drees et al., 1995*, we have now constructed high-copy number plasmids with Tpm1 or Tpm2 expression driven under the galactose-inducible *GAL1* promoter (pYES2-GAL1-Tpm1/2). We tested growth rescue of *∆tpm1* cells under three different galactose concentrations - No Galactose, 2% Galactose, and 4% Galactose. We observe a complete rescue of growth at all temperatures tested by Tpm1 and Tpm2 expression in the presence of 2% and 4% Galactose, as compared to cells carrying empty pYES2-GAL1 plasmid. We also observed a partial rescue of growth in cells expressing only Tpm1 or Tpm2 containing plasmid in the absence of galactose, which is most probably caused by leaky expression of the GAL1 promoter. These observations further corroborate our results and demonstrate that increase in Tpm2 levels can restore normal growth and actin cables in *∆tpm1* cells. These results suggest that there may be other reasons such as mutations in plasmid construct or strain background, which may have led to contrasting conclusions about Tpm2 functionality in *Drees et al., 1995*. We have now included and discussed these results as new **Fig. S6** in the revised manuscript **(Lines 281-291)**.

5. **Clarity and context**  · For Figure 2A and Figure 2D, it would be helpful to use arrows to point to obvious Tpm-coated cables since the Tpm1 constructs have high cytosolic levels.

 As per the reviewers’ suggestions, we have now used an inverted gray LUT to better represent the actin cables in **Fig. 2A, 2D** and used arrows to point to Tpm-bound actin cables.

· Throughout the paper, different plasmids are used to express combinations of Tpm isoforms and promoters. It would be extremely helpful for the readers if the group could create a legend with simplified descriptions of the plasmids properties (i.e. high expression, low expression) and associating these labels when the plasmids are mentioned. Including these simplified labels in the figures would allow the reader to quickly understand what is different between pRS backbones.

We thank the reviewer's excellent suggestion to improve the clarity of our plasmid nomenclature. To address this, we have implemented a consistent and simplified naming system throughout the manuscript, including all figures.

In this system, we add a subscripted letter in parentheses to the *pRS* backbone name to indicate the plasmid's copy number:

**(L)** for low-copy (e.g. *pRS316*), **(H)** for high-copy (e.g. *pRS425*), and **(I)** for integration (e.g. *pRS305*). This allows readers to immediately understand the plasmid's properties. For example, a low-copy plasmid expressing Tpm1 is now referred to as *pRS316_(L)_-Tpm1.*

We have also created a dedicated legend in the Materials and Methods section **(Lines 560-567)** that describes this system and lists all plasmids used in the study. This will enable readers to quickly and easily understand the properties and expression levels of the constructs used in each experiment.
